# Effects of *Ampelopsis brevipedunculata* on Ovalbumin-Induced Allergic Airway Inflammation via Regulating Mast Cell Activation and PLCγ/Akt/NF-κB Signaling Pathway

**DOI:** 10.3390/molecules31193479

**Published:** 2026-09-29

**Authors:** Yeon-Yong Kim, Hyun-Jae Jang, Seungwon Jeong, Seung Woong Lee, Sang-Hyun Kim, Soyoung Lee

**Affiliations:** 1 Functional Biomaterial Research Center, Korea Research Institute of Bioscience and Biotechnology (KRIBB), 181, Ipsin-gil, Jeongeup 56212, Republic of Korea; saxyyou12@gmail.com (Y.-Y.K.); water815@kribb.re.kr (H.-J.J.); lswdoc@kribb.re.kr (S.W.L.); 2 Department of Bionanotechnology and Bioconvergence Engineering, Jeonbuk National University, 567, Baekje-daero, Jeonju 54896, Republic of Korea; uka167@jbnu.ac.kr; 3 Department of Innovative Pharmaceutical Sciences, Advanced Institute of Science and Technology, Kyungpook National University, 80, Daehak-ro, Daegu 41566, Republic of Korea; 4 Cell Matrix Research Institute, Department of Pharmacology, School of Medicine, Kyungpook National University, 680, Gukchaebosang-ro, Daegu 41944, Republic of Korea

**Keywords:** *Ampelopsis brevipedunculata* (Maxim.) Trautv., allergic asthma, airway inflammation, mast cell, degranulation

## Abstract

Asthma is a chronic allergic airway inflammation that requires ongoing therapeutic intervention and reduces the patient’s quality of life. Therefore, safe and efficacious drugs for the treatment of asthma are urgently needed. This study investigated whether the ethyl acetate fraction derived from a 95% (*v*/*v*) ethanol extract of dried *Ampelopsis brevipedunculata* (ABE) alleviates allergic airway inflammation. To investigate the anti-allergic and anti-inflammatory effects of ABE, a mouse model of ovalbumin (OVA)-induced allergic airway inflammation was used and clarified their mechanisms in mast cells. ABE administration reduced the asthmatic symptoms and levels of pro-inflammatory mediators and cytokines by 45–70% in bronchoalveolar lavage fluid (BALF) and by 55–80% in lung tissue compared with the OVA group. ABE administration decreased the levels of serum immunoglobulins (Igs) including IgG1, IgG2a, IgE, and OVA-specific IgE by 30–55% compared with the OVA group. In addition, ABE ameliorated mast cell infiltration and activation which reflected the degree of allergic airway inflammation in the lung and BALF. ABE inhibited mast cell degranulation, suppressing intracellular calcium influx by approximately 30% relative to the DNP-HSA group. It also prevented pro-inflammatory gene expression of mast cells by 35–70% relative to the DNP-HSA group through blocking the FcεRI-NF-κB signaling pathway. Moreover, the isolated compounds from ABE (syringic acid, ellagic acid, resveratrol, catechin, and 4′-*O*-(β-*D*-glucopyranosyl)-3,3′4-tri-*O*-methylellagic acid) inhibited mast cell degranulation and pro-inflammatory cytokine expression by suppressing the PLCγ, Akt, and NF-κB signaling pathways. Taken together, these findings suggest that ABE and its constituents are potential novel therapeutic agents for allergic airway inflammation.

## 1. Introduction

Asthma is a prevalent airway disease and a chronic respiratory disease induced by allergic reactions in the airway. It is characterized by shortness of breath, wheezing, and cough [1]. Allergy is a hypersensitive response of the immune system triggered by normally innocuous environmental substances such as pollen, dust, mites, and certain proteins and other chemicals [2]. Airway inflammation frequently occurs in asthma. The main symptoms of allergic airway inflammation are airway constriction, airway smooth muscle hyperreactivity, airflow obstruction, goblet cell hyperplasia, mucus hypersecretion, and collagen deposition. These manifestations cause damage to the airway and impair lung function, resulting in severe asthma [3]. It is promoted by allergic reactions and immune cell activation and may result in permanent airway remodeling and diminished quality of life [4].

Allergic asthma is initiated by activating allergen-specific T-helper type 2 (Th2) cells with antigen-presenting cells (APCs). Antigen-exposed APCs trigger naïve CD4^+^ T-cell activation which, in turn, induces the differentiation of helper T-cells such as Th1, Th2, Th17, and Treg. These processes occur with the activation of immunocytes such as eosinophils, macrophages, neutrophils and mast cells [5,6]. Mast cells play central roles in allergic reactions and inflammatory responses and are associated with Th2 responses. They occur in the skin, mucosae, lungs, intestines, and the nervous system. They release potent immune mediators such as histamines, proteases, chemotactic factors, cytokines, and arachidonic acid metabolites [7,8]. These substances act on the vasculature, smooth muscle, connective tissue, mucous glands, and inflammatory cells and initiate bronchoconstriction, leukocyte infiltration and activation, and tissue remodeling in allergic airway inflammation [9,10,11].

Currently approved treatment strategies for mast cell-mediated diseases include the use of anti-immunoglobulin (Ig) E monoclonal antibodies (mAbs) such as omalizumab and histamine H1 receptor antagonists (anti-histamines). Although anti-IgE mAbs have the advantage of blocking receptor cross-linking, they dissociate IgE already bound to the mast cell surface at a slow rate. Therefore, repeated administration is essential to maintain therapeutic effects. In the case of anti-histamines, which are representative allergy symptom relievers, there is a limitation in that they have little effect even when administered at the early stages of an allergic reaction [12]. On the other hand, inhaled glucocorticoids such as dexamethasone (DEX) are widely used to treat asthma to suppress airway inflammation, even though they do not specifically target mast cells. However, they are associated with side effects including metabolic disorders, gastrointestinal and cardiovascular effects [13]. For this reason, efficacious therapeutic strategies against mast cell-mediated airway inflammation are urgently required.

Natural products exhibit structural characteristics distinct from synthetic compounds, featuring enhanced molecular rigidity. These properties have provided significant advantages for the discovery of new drugs targeting protein–protein interactions. Furthermore, the use of natural products in traditional medicine offers potential for efficacy and safety [14]. *Ampelopsis brevipedunculata* (Maxim.) Trautv. is a vine harvested in Asia, North America, Mexico, and Guatemala. *Ampelopsis brevipedunculata* has been used in traditional medicine to treat various diseases and has antioxidant and anti-hepatotoxicity efficacy [15,16]. We previously reported that the ethyl acetate fraction of a 95% ethanol extract of *A. brevipedunculata* vines (ABE) and its isolated bioactive compounds suppressed IL-6-induced STAT3 activation [17]. Recent studies have demonstrated the potent anti-allergic and anti-inflammatory properties of ABE. Specifically, ABE rhizome ethanol extract was reported to suppress IgE-mediated mast cell activation and systemic anaphylaxis [18]. In another recent study, it was demonstrated that ABE had anti-dermatitis efficacy by inhibiting the STAT1/MAPKs signaling pathways in keratinocytes which, in turn, suppressed T helper cell polarization and immune responses [19]. Despite these findings on mast cell degranulation and skin allergic responses, the therapeutic potential of ABE and the underlying molecular mechanism in allergic airways have not yet been established. Moreover, it remains unclear which specific active constituents of ABE contribute to its inhibitory effects on allergic inflammation. To address this unmet need, we investigated the therapeutic efficacy of ABE and its constituents in IgE-mediated mast cell activation and in an in vivo model of allergic airway inflammation, demonstrating their potential as novel therapeutic agents for allergic asthma.

## 2. Results

### 2.1. Effects of ABE on OVA-Induced Allergic Airway Inflammation in Mice

**Figure 1:**
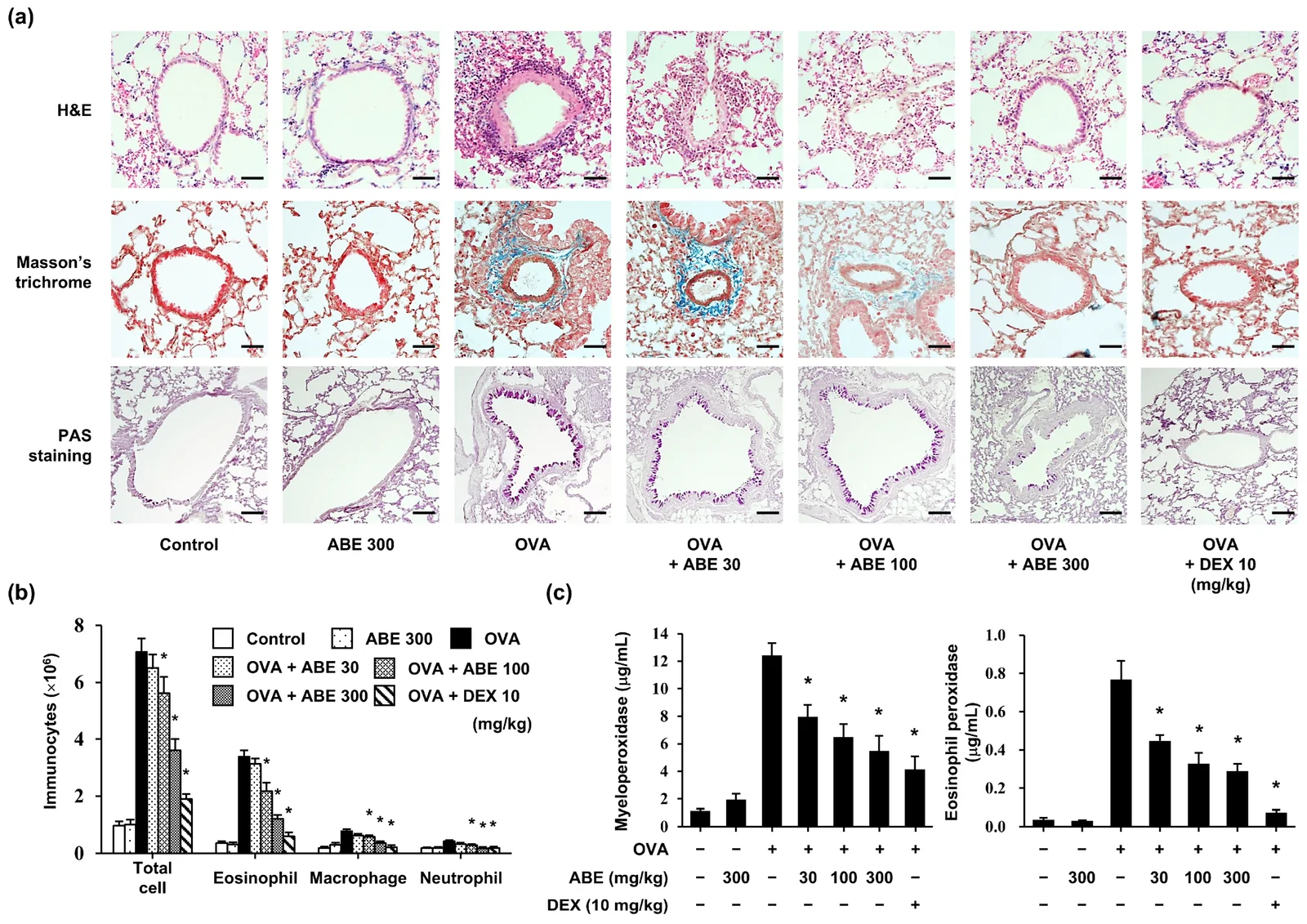
Effects of ABE on an OVA-induced airway inflammation model. The histological pathology was observed in the left lobes of the lung. (**a**) Representative images of H&E, Masson’s trichrome and PAS-stained lung sections (×200 magnification, scale bar (black) size: 100 μm). (**b**) Differential counts of Diff-Quik-stained immune cells in BALF. (**c**) MPO and EPO levels in BALF were measured by ELISA. Dexamethasone (DEX) served as a positive control. Data points represent the mean ± SEM (*n* = 8 per group). Statistical significance was analyzed using one-way ANOVA followed by Dunnett’s test. * *p* < 0.05. relative to the OVA group.

**Figure 2:**
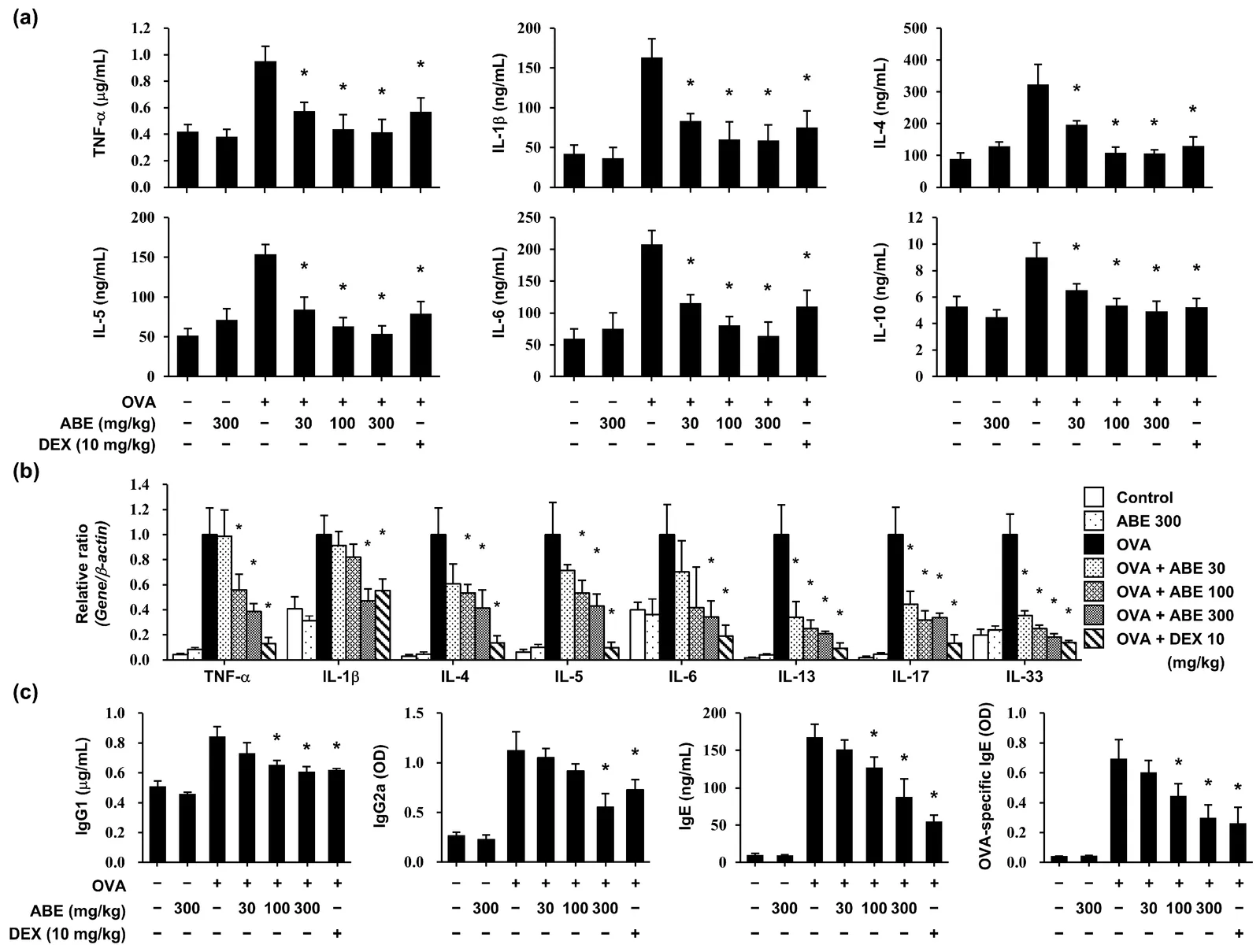
Effects of ABE on pro-inflammatory gene expression in OVA-induced airway inflammation model mice. Pro-inflammatory and Th2 cytokine levels were determined by ELISA and qPCR. (**a**) Cytokine levels were measured in BALF supernatant. (**b**) Gene expression was evaluated in homogenates of the right superior lobe of the lung. (**c**) The serum Ig levels were measured in blood obtained by heart puncture from each mouse. Collected blood was immediately centrifuged at 400× *g* and 4 °C for 10 min, and the upper layer was analyzed for Ig levels by ELISA. Dexamethasone (DEX) was used as a positive control. Data points represent the mean ± SEM (*n* = 8 per group). Statistical significance was analyzed using one-way ANOVA followed by Dunnett’s test. * *p* < 0.05. relative to the OVA group.

To assess the anti-asthmatic efficacy of ABE, we examined histological changes in the lung tissue of OVA-induced airway inflammatory mice. ABE administration ameliorated OVA-challenged airway constriction, collagen fiber accumulation, and goblet cell hyperplasia (Figure 1a). ABE administration also attenuated the OVA-induced increase in Muc5ac expression, as assessed by immunohistochemistry (Figure S1). To confirm the effects of ABE on immune cell infiltration in the lung, we counted the stained immune cells in BALF. ABE treatment attenuated relative OVA-induced increases in total immune cells, eosinophils, macrophages, and neutrophils (Figure 1b). Moreover, it mitigated OVA-induced elevations in MPO and EPO (Figure 1c). Our mouse model showed that ABE administration inhibited OVA-challenged upregulation of pro-inflammatory (TNF-α, IL-1β, IL-6, and IL-17) and Th2 (IL-4, IL-5, IL-10, IL-13, and IL-33) cytokines in BALF and lung tissue (Figure 2a,b). ABE treatment counteracted OVA-challenged serum IgG1, IgG2a, IgE, and OVA-specific IgE upregulation (Figure 2c). Our data suggested that ABE repressed allergic airway inflammation.

### 2.2. Effects of ABE on Mast Cell Activation in Murine OVA-Induced Allergic Airway Inflammation

**Figure 3:**
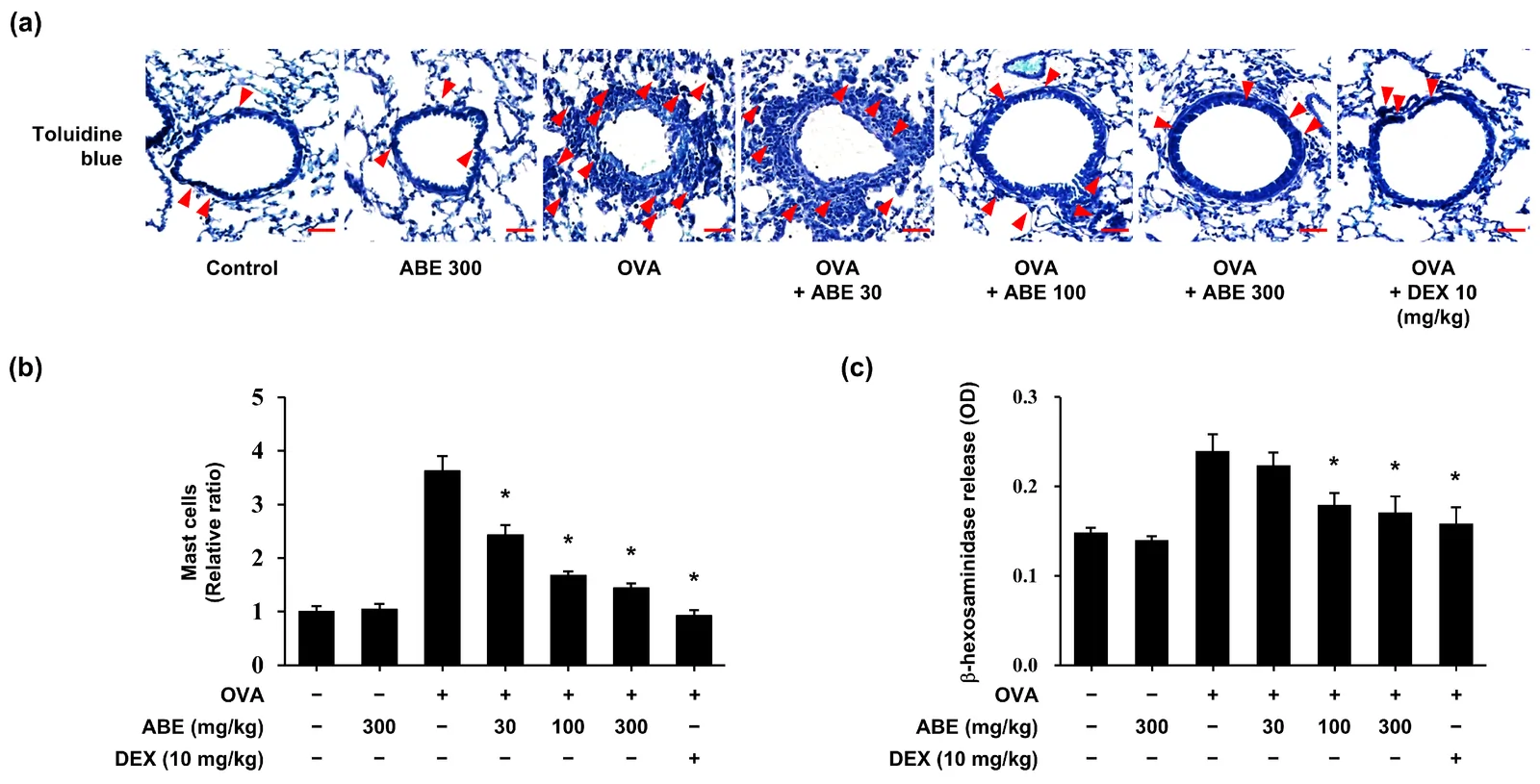
Effects of ABE on mast cell activation in OVA-induced allergic airway inflammation model mice. (**a**) Representative images of toluidine blue-stained lung sections (×200 magnification, scale bar (red) size: 100 μm). The mast cells were stained deep blue (indicated by the red arrow). (**b**) Enumeration of mast cells in a toluidine blue-stained lung section. (**c**) Evaluation of β-hexosaminidase level in BALF supernatant. Dexamethasone (DEX) was used as a positive control. Data points represent the mean ± SEM (*n* = 8 per group). Statistical significance was analyzed using one-way ANOVA followed by Dunnett’s test. * *p* < 0.05. relative to the OVA group.

To determine whether mast cells were implicated in the mouse OVA-induced airway inflammatory model, we examined mast cell infiltration into lung tissue by toluidine blue staining. ABE treatment suppressed OVA-challenged increases in mast cell infiltration into the lung (Figure 3a,b). Similarly, immunohistochemical staining results revealed that ABE treatment attenuated the OVA-induced increase in FcεR1α (Figure S2). ABE treatment also reduced OVA-induced β-hexosaminidase release (Figure 3c).

### 2.3. Effects of ABE on Mast Cell Degranulation In Vitro

**Figure 4:**
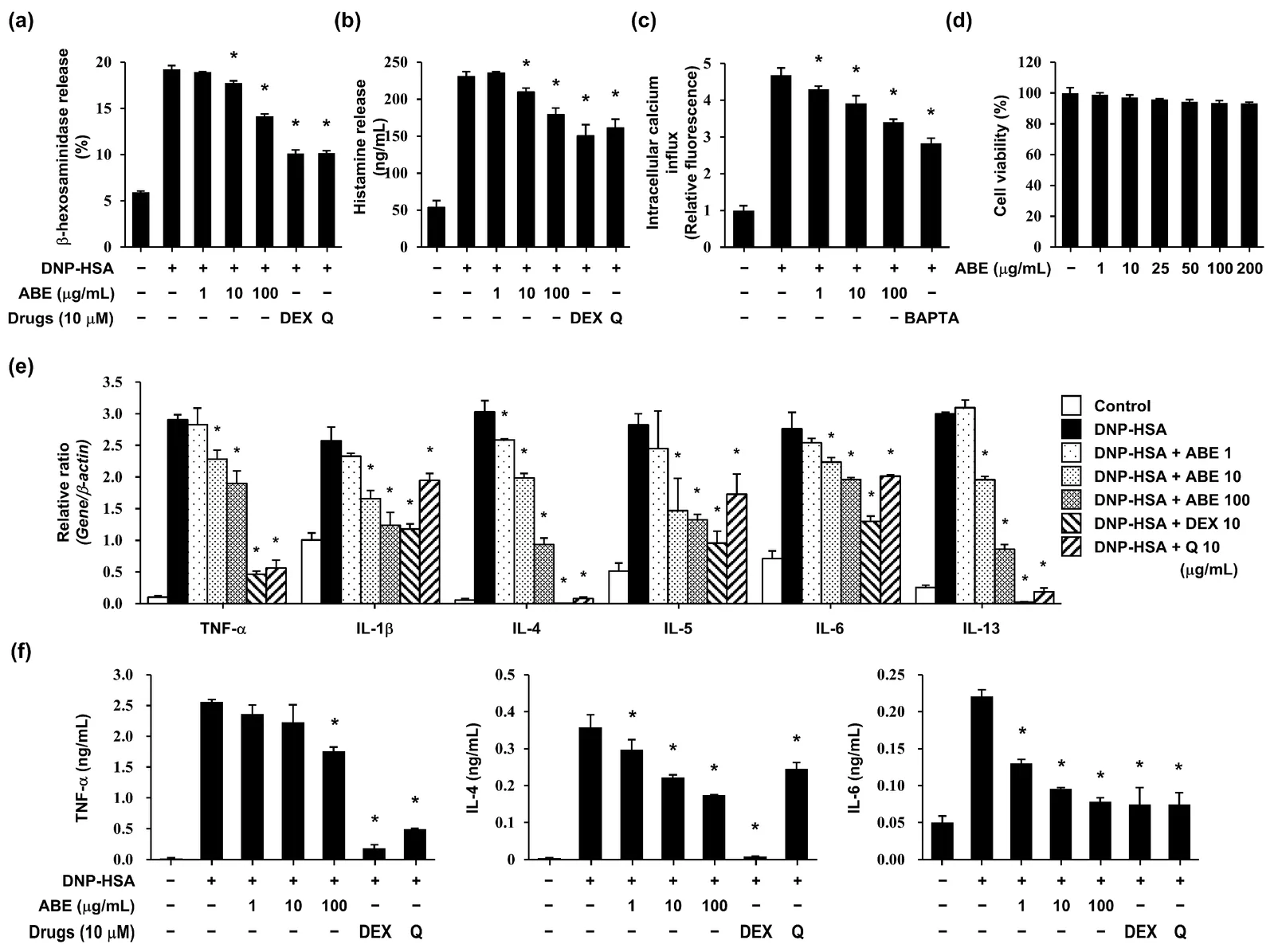
Effects of ABE on mast cell degranulation and pro-inflammatory cytokine expression. The assessment of mast cell activation was performed on RBL-2H3 cell lines. The cells were sensitized with anti-DNP-IgE and then challenged with DNP-HSA. (**a**) β-hexosaminidase levels were determined by spectrophotometry. (**b**) Histamine levels were detected in a fluorescence plate reader. (**c**) RBL-2H3 cells were preincubated with Fluo-3/AM. Intracellular calcium was measured with a fluorescence plate reader. The calcium chelator BAPTA served as a positive control. (**d**) ABE cytotoxicity on RBL-2H3 cells was evaluated by MTT assay. The pro-inflammatory and Th2 cytokine expression profile of mast cells was performed on RBL-2H3 cell lines. The cells were sensitized with anti-DNP-IgE, and then challenged with DNP-HSA. The cytokine expression (**e**) and secretion (**f**) were measured by qPCR and ELISA respectively. Dexamethasone (DEX) and Quercetin (Q) were used as positive controls. Data points represent the mean ± SEM (*n* = 3 per group). Statistical significance was analyzed using one-way ANOVA followed by Dunnett’s test. * *p* < 0.05. relative to the DNP-HSA group.

To determine the anti-allergic effects of ABE, a mast cell degranulation assay was performed in vitro. ABE pretreatment inhibited DNP-HSA-challenged mast cell degranulation such as β-hexosaminidase and histamine release in a concentration-dependent manner (Figure 4a,b). To elucidate the mechanism by which ABE suppresses mast cell degranulation, we used the fluorescence indicator Fluo-3/AM to measure intracellular calcium in DNP-HSA-challenged mast cells. ABE pretreatment attenuated DNP-HSA-induced increases in intracellular calcium levels in a concentration-dependent manner (Figure 4c). The MTT assay indicated that ABE was not cytotoxic in the concentration range of 200 μg/mL (Figure 4d). To determine the anti-inflammatory effects of ABE, we evaluated pro-inflammatory gene expression and cytokine secretion. ABE administration suppressed DNP-HSA-induced upregulation of the pro-inflammatory cytokines (TNF-α, IL-1β, and IL-6) and Th2 cytokines (IL-4, IL-5, and IL-13) in a concentration-dependent manner (Figure 4e). Furthermore, it inhibited DNP-HSA-challenged increases in cytokines including TNF-α, IL-4, and IL-6 (Figure 4f).

### 2.4. Effects of ABE on Mast Cell Signaling Pathways

**Figure 5:**
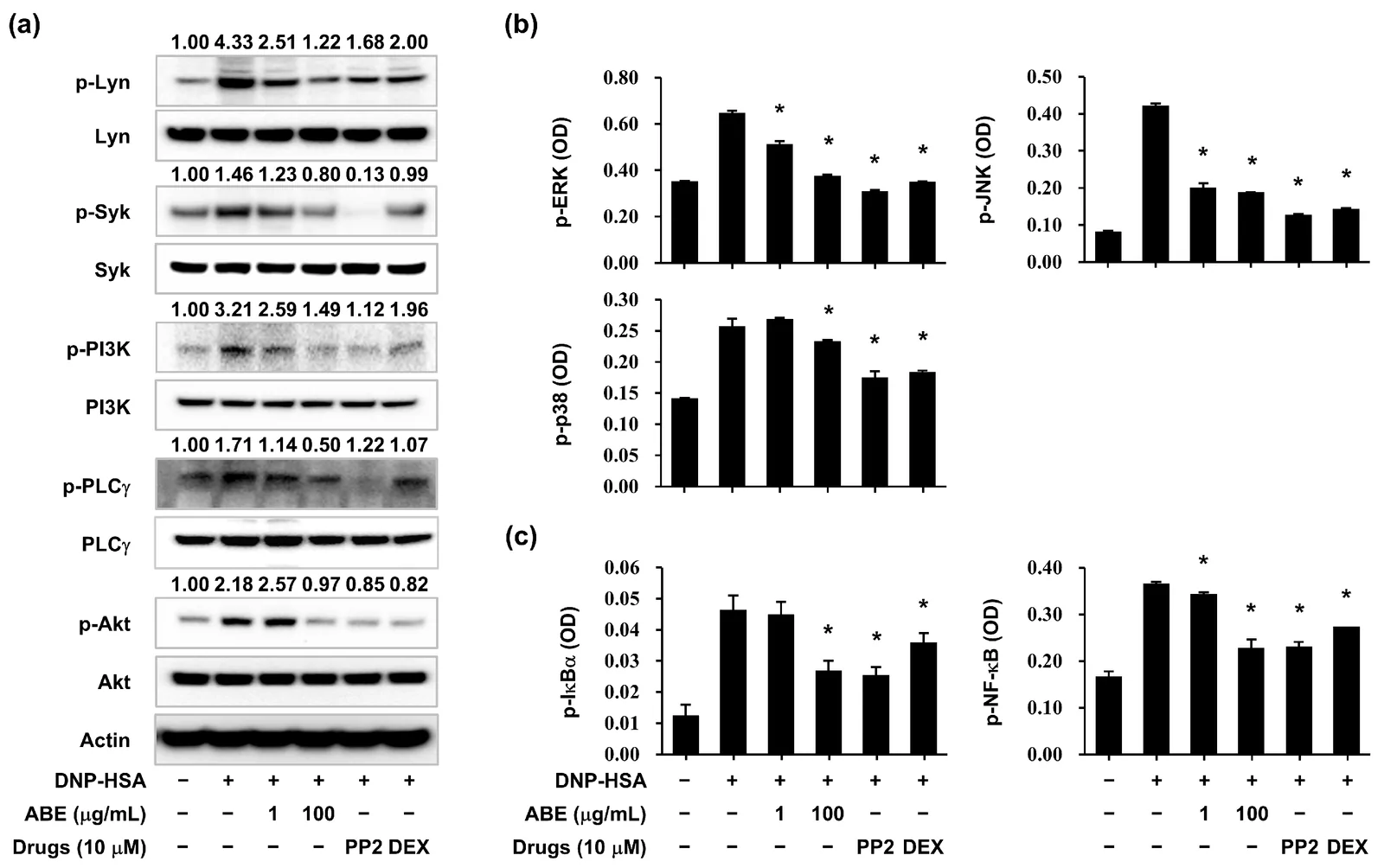
Effects of ABE on mast cell signaling pathways. Protein samples were extracted from whole RBL-2H3 cells. (**a**) Phosphorylation of mast cell signaling pathway factors such as Lyn, Syk, PI3K, PLCγ, and Akt was measured by Western blotting. Phosphorylation of MAPKs such as ERK, JNK, and p38 (**b**) and IκBα and NF-κB (**c**) was analyzed by ELISA. The Src kinase inhibitor PP2 and dexamethasone (DEX) served as positive controls. Data points represent the mean ± SEM (*n* = 3 per group). Statistical significance was analyzed using one-way ANOVA followed by Dunnett’s test. * *p* < 0.05. relative to the DNP-HSA group.

To clarify the mechanisms by which ABE inhibits mast cell degranulation and pro-inflammatory gene expression, we analyzed mast cell signaling pathways by using Western blot and ELISA. Western blot showed that ABE pretreatment suppressed DNP-HSA-induced phosphorylation of mast cell signaling pathway factors such as Lyn, Syk, PI3K, PLCγ, and Akt (Figure 5a). ABE treatment blocked DNP-HSA-challenged MAPKs (Figure 5b), IκBα, and NF-κB protein phosphorylation (Figure 5c). To further investigate the downstream signaling cascades by which ABE suppresses degranulation and inflammation, we evaluated the nuclear translocation of transcription factors, including NFAT1 and NF-κB. DNP-HSA stimulation significantly increased the nuclear levels of both NFAT1 and NF-κB compared to the control group. Moreover, the cytosolic levels of NFAT1 were decreased by DNP-HSA stimulation. However, ABE treatment reduced translocation of these transcription factors into the nucleus (Figure S3). These findings demonstrate that ABE effectively suppresses mast cell activation by modulating the phosphorylation signaling pathway and preventing the subsequent nuclear translocation of NFAT1 and NF-κB.

### 2.5. Effects of Isolated ABE Compounds on Mast Cell Activation

**Figure 6:**
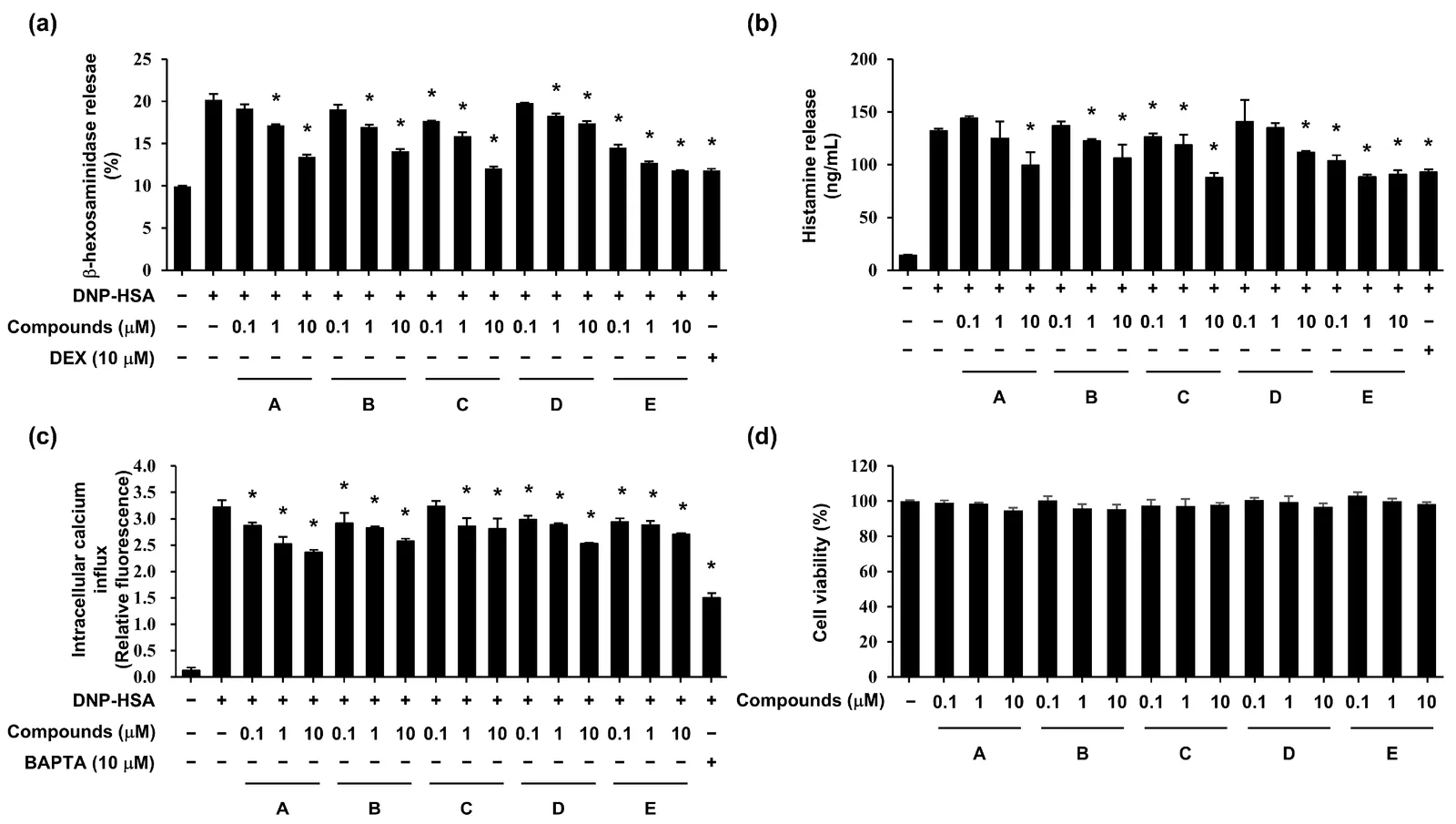

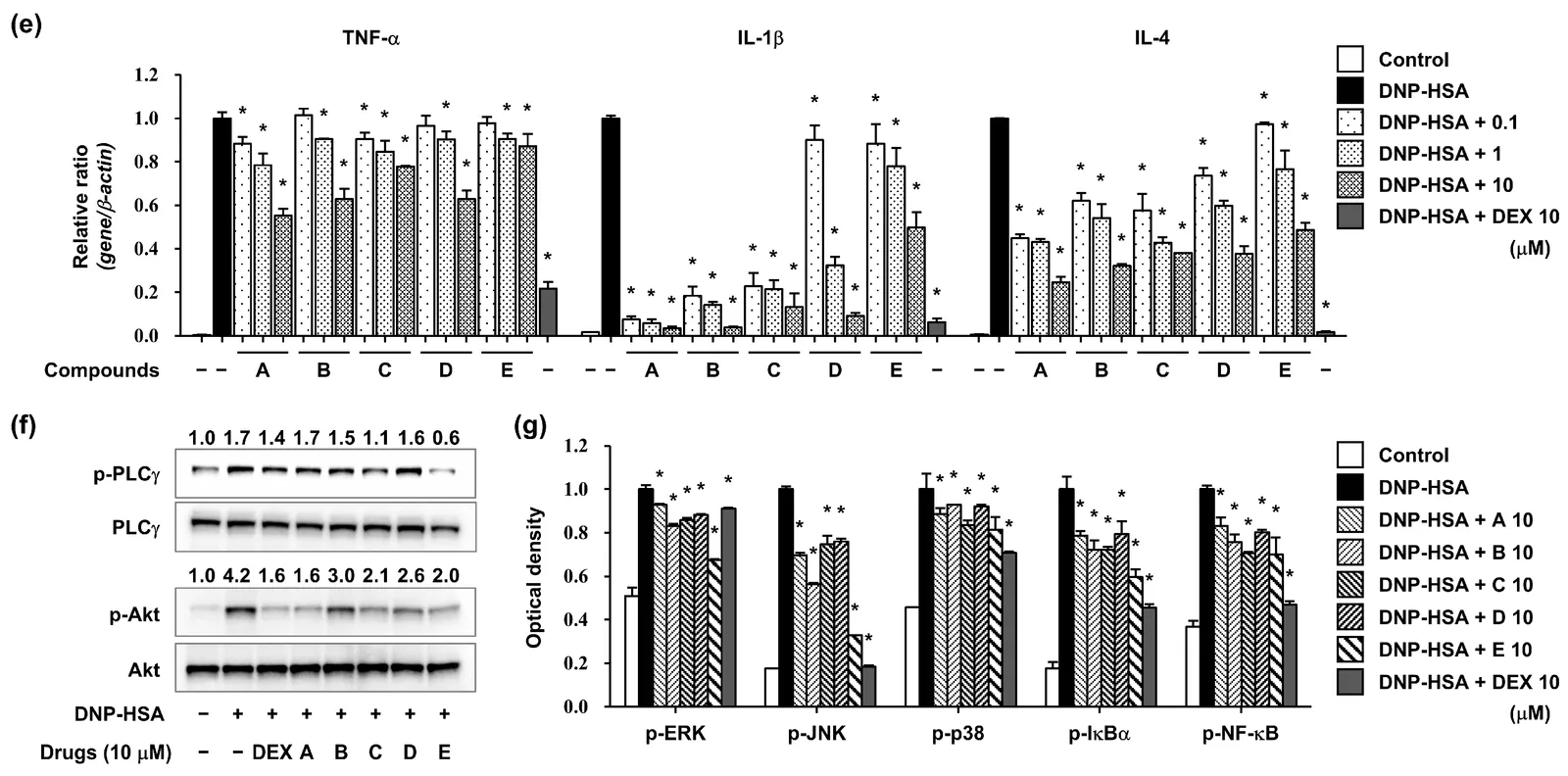
Effect of isolated ABE compounds on mast cell degranulation and activation. Anti-allergic efficacies of isolated ABE compounds on DNP-HSA-challenged mast cells were determined. (**a**) β-hexosaminidase levels were measured by spectrophotometry. (**b**) Histamine levels were detected with a fluorescence plate reader. (**c**) Intracellular calcium was evaluated using a fluorescence plate reader. The calcium chelator BAPTA served as a positive control. (**d**) Cytotoxicity of isolated compounds on RBL-2H3 cells was assessed by MTT assay. The five compounds did not show cellular toxicity in the experimental concentration range (0.1–10 μM). Anti-inflammatory properties and mechanisms of isolated ABE compounds on DNP-HSA-induced mast cells were determined. (**e**) Pro-inflammatory cytokine gene expression was measured by qPCR. (**f**) PLCγ and Akt phosphorylation were evaluated by Western blot. (**g**) Phosphorylation of ERK, JNK, p38, IκBα, and NF-κB was analyzed by ELISA. A, syringic acid; B, ellagic acid; C, resveratrol; D, catechin; E, 4′-*O*-(β-*D*-glucopyranosyl)-3,3′4-tri-*O*-methylellagic acid. Dexamethasone (DEX) served as a positive control. Each data point represents the mean ± SEM (*n* = 3 per group). Statistical significance was analyzed using one-way ANOVA followed by Dunnett’s test. * *p* < 0.05. relative to the DNP-HSA group.

To identify the active compounds having an anti-allergic efficacy of ABE, ABE was isolated into single compounds. We evaluated the inhibitory effects of the 17 isolated single compounds on mast cell degranulation (Table S2, Figure S4) and we selected five active compounds A–E (syringic acid, ellagic acid, resveratrol, catechin, and 4′-*O*-(β-*D*-glucopyranosyl)-3,3′4-tri-*O*-methylellagic acid). The chemical structures of these five active compounds are shown in Figure S5. These five compounds were selected because they constitute the most abundant active components of ABE (Compound D) or demonstrate effective inhibitory efficacy (Compounds A, B, C and E) comparable to that of DEX in preliminary screening assays for mast cells. Pretreatment with these five isolated compounds reduced DNP-HSA-challenged mast cell degranulation (Figure 6a,b) by inhibiting intracellular calcium influx (Figure 6c). None of the five compounds were cytotoxic at the experimental concentration range (Figure 6d). In addition, pretreatment with these substances inhibited DNP-HSA-induced pro-inflammatory cytokine expression (Figure 6e). It also suppressed DNP-HSA-challenged activation of mast cell signaling pathway factors such as PLCγ and Akt (Figure 6f). Moreover, it alleviated DNP-HSA-challenged MAPK, IκBα, and NF-κB protein phosphorylation (Figure 6g).

## 3. Discussion

Mast cells have been studied to play various roles in lung disease as well as allergic reactions. Mast cells contribute to idiopathic pulmonary fibrosis by stimulating airway smooth muscle [20], and to acute lung injury by releasing proteases, ROS, and peptides [21]. A recent study showed that SARS-CoV-2 infection-mediated mast cell activation exacerbated acute severe lung inflammation via a cytokine storm in the airway [22]. In asthma, mast cells are key effectors in bronchoconstriction and airway remodeling [23]. Thus, lung disease research based on mast cells will provide a new therapeutic perspective.

In the OVA-induced mouse model established to investigate the pathogenesis of allergic asthma, OVA sensitization enhances IgE secretion from B cells, and then OVA re-exposure initiates an allergic response through the binding of antigens to IgE-FcεRI receptor complexes on mast cell surfaces [7,24]. The activated mast cells immediately release histamine and inflammatory cytokines, causing bronchoconstriction, mucosal edema, and mucus hypersecretion and persistent airway inflammation [25,26]. Furthermore, OVA stimulation significantly increased mast cell infiltration and serum IgE, IgG1, and IgG2a levels. The overexpression of these Igs and inflammatory cytokines is a key indicator determining disease severity [27,28]. In this study, ABE administration dose-dependently alleviated these pathological responses, demonstrating that ABE simultaneously controls both the initial initiation and progression phases of allergic inflammation. Notably, ABE administration not only suppressed OVA-induced mast cell infiltration into the lung but also attenuated FcεR1α expression and β-hexosaminidase release, indicating that ABE inhibits mast cell activation. In addition, ABE administration reduced the infiltration of eosinophils, neutrophils, and macrophages within the BALF, as well as cytokine and serum Ig levels. Taken together, these results indicate that ABE integrally suppresses local airway inflammation and systemic allergic sensitization. Furthermore, the improvement in histopathology confirmed by H&E, PAS, and MT staining demonstrates that ABE possesses structural protective value. This effect was manifested by preventing both inflammatory cell infiltration into lung tissue and airway obstruction caused by goblet cell hyperplasia and subepithelial collagen deposition. This protective effect was further supported by the reduction in Muc5ac expression, a predominant mucin involved in mucus hypersecretion in airway epithelium [29]. These in vivo results suggest that ABE possesses pharmacological potential as a promising natural therapeutic candidate for management of allergic asthma by regulating mast cell activation and immune responses.

We used the RBL-2H3 mast cell line to evaluate the anti-allergic inflammatory mechanism of ABE. The RBL-2H3 cells were activated by cross-linking antigen, IgE, and the high-affinity IgE receptor FcεRI expressed on their surfaces. This process is associated with an intracellular signaling pathway. Antigen-IgE-FcεRI cross-linkage triggers Src-family protein tyrosine kinase (Lyn and Syk) phosphorylation. These proteins activate cascade reactions including PI3K, PLCγ, Akt, MAPKs, IκBα, and NF-κB. Phosphorylated PLCγ mediates calcium ion release from the endoplasmic reticulum and leads to the release of β-hexosaminidase and histamine [27,30]. β-hexosaminidase secretion is a mast cell degranulation factor [31]. Histamine triggers immediate allergic responses including vascular permeability, vasodilation, bronchoconstriction, and hypothermia [32]. In our study, a DNP-HSA challenge induced mast cell degranulation, an increase in intracellular calcium levels, and PLCγ phosphorylation. However, all the foregoing effects were suppressed by ABE. Previous reports indicated that intracellular calcium increases histamine release [33,34]. Thus, calcium is an important secondary messenger that indicates mast cell degranulation. Elevated intracellular calcium also activates calcineurin, which dephosphorylates NFAT1 and promotes its translocation into the nucleus, thereby driving the transcription of inflammatory cytokine genes [35,36]. In the present study, DNP-HSA challenge increased nuclear NFAT1 levels, and this increase was suppressed by ABE treatment. The preceding results suggest that the anti-degranulation effect of ABE is associated with intracellular calcium reduction via the inhibition of PLCγ phosphorylation. In addition, the present study showed that Akt and MAPKs were triggered by the DNP-HSA challenge. Akt plays a vital role in inflammatory signaling progression [37]. Phosphorylated Akt activates IκBα phosphorylation which, in turn, initiates nuclear NF-κB translocation and the expression of several genes involved in inflammatory responses [7]. MAPKs also participate in pro-inflammatory gene expression mediated by the PI3K signaling pathway [38]. The present study demonstrated that DNP-HSA challenge induced the phosphorylation of key signaling molecules involved in FcεRI-mediated mast cell activation, including Lyn, Syk, PI3K, Akt, MAPKs, IκBα, and NF-κB. Notably, ABE treatment attenuated the phosphorylation of these signaling molecules and further suppressed the nuclear translocation of both NFAT1 and NF-κB. Taken together, these findings indicate that the anti-allergic inflammatory effects of ABE are mediated through the suppression of FcεRI downstream signaling pathways in mast cells. Thus, ABE influences the anti-allergic airway inflammatory effect by regulating mast cell activation.

We demonstrated and confirmed the anti-allergic inflammatory effect of ABE on an OVA-induced allergic airway inflammation model and mast cell activation. To find the specific ABE constituents with these properties, we isolated individual compounds from *A. brevipedunculata* using HPLC/MPLC and selected five with anti-degranulation activity. These included syringic acid, ellagic acid, resveratrol, catechin, and 4′-*O*-(β-*D*-glucopyranosyl)-3,3′4-tri-*O*-methylellagic acid. According to previous studies, these compounds have been reported to inhibit intracellular calcium influx and downregulate pro-inflammatory genes by regulating the PLCγ, Akt, MAPK and NF-κB signaling pathways. Specifically, syringic acid alleviates inflammatory responses by inhibiting PI3K, Akt, and NF-κB pathways [39], while ellagic acid exhibits anti-inflammatory properties and suppresses intracellular calcium mobilization by regulating PLCγ, PKC, MAPKs, and Akt [40]. Additionally, resveratrol and catechin have demonstrated outstanding anti-inflammatory effects in various inflammatory disease models by blocking MAPKs, PI3K, Akt, and NF-κB signaling pathways [41,42,43]. In particular, these compounds play a role in suppressing mast cell activation. Syringic acid effectively inhibited mast cell infiltration into IgE-sensitization sites in passive cutaneous anaphylaxis (PCA) mouse models [44], and ellagic acid and resveratrol inhibited the pigmentation of extravasation in PCA rat models and suppressed mast cell degranulation by suppressing calcium influx and NF-κB translocation in rat peritoneal mast cells [45]. Moreover, a recent study investigated the inhibitory effect of resveratrol on IL-33 in ST2-mediated mast cell activation [46]. Catechin is a polyphenolic compound and has shown inhibitory effects on compound 48/80-stimulated calcium influx and degranulation of mast cells [47]. These studies and our results indicate that ABE has anti-allergic inflammatory effects through the integrated biological effects of its constituents. Although 4′-*O*-(β-*D*-glucopyranosyl)-3,3′4-tri-*O*-methylellagic acid has seldom been evaluated [48], our study showed that this molecule strongly inhibited mast cell degranulation and signaling transductions including PLCγ, Akt, MAPKs and NF-κB. Therefore, ABE and these compounds may be candidates as therapeutic agents in the treatment of mast cell-mediated allergic airway inflammation.

This study demonstrated the efficacy of ABE on mast cell-mediated allergic airway inflammation. However, the OVA-induced allergic airway inflammation mouse model represents a classical allergic inflammation model [49], which lacks the capacity to mimic the pathological features of clinically established chronic asthma such as persistent airway remodeling. Therefore, further studies will be necessary to evaluate the therapeutic efficacy of ABE in a chronic airway inflammation model induced by long-term OVA exposure. Furthermore, while oral administration of ABE demonstrated a clear dose-dependent protective effect in the mouse model used in this study, several considerations are required when extrapolating these mouse doses to humans. Due to significant species differences in factors such as gastrointestinal absorption, hepatic first-pass metabolism, and systemic pharmacokinetic profiles, converting animal doses to human doses based solely on body surface area (BSA) serves only as a preliminary indicator. Based on studies to date, comprehensive pharmacokinetic (PK) data for the crude ABE extract as well as specific components isolated from ABE (compounds A, B, C, and E) are virtually non-existent. While the pharmacokinetic profile of catechin (compound D) is well-established [50], systemic pharmacokinetic parameters for the ABE extract as a whole and for the other active compounds have not yet been defined. Therefore, future studies must include detailed in vivo pharmacokinetic and pharmacodynamic (PK/PD) evaluations to clearly elucidate their absorption, distribution, metabolism, and excretion (ADME) pathways and to establish optimal therapeutic regimens for clinical application. In addition, our data show that ABE suppresses the phosphorylation of early signaling kinases (Lyn and Syk), as well as downstream signaling events in both the PLCγ–Ca^2+^–NFAT1 and PI3K/Akt pathways. Given the short treatment period (1 h pretreatment followed by 7–30 min of stimulation) and the unchanged total levels of Lyn, Syk, PI3K, PLCγ, and Akt (Figure 5a), these effects are most likely due to reduced signal activation rather than altered protein expression. However, total and phosphorylated levels of Gab2, a scaffolding adaptor that links FcεRI activation to PI3K signaling [51], were not determined in the present study; therefore, a possible contribution of altered Gab2 abundance to the observed effects cannot be completely excluded. Further studies directly assessing Gab2 abundance and phosphorylation, as well as Fyn activation, will help to fully clarify the molecular mechanism by which ABE inhibits FcεRI signaling in mast cells.

## 4. Materials and Methods

### 4.1. Preparation of ABE and Isolation of Its Constituents

#### 4.1.1. Plant Material and Preparation of the Ethyl Acetate Fraction

The vines of *A. brevipedunculata* (Maxim.) Trautv. (Vitaceae) were purchased from an herbal store in Seoul, Republic of Korea, in May 2014. Botanical identification was performed by Dr. Rho of the Korea Research Institute of Bioscience & Biotechnology (KRIBB). The scientific name was verified against World Flora Online (WFO), in which *A. brevipedunculata* (Maxim.) Trautv. (WFO ID: wfo-0000531629) is treated as a synonym of the accepted name *Ampelopsis glandulosa var. brevipedunculata* (Maxim.) Momiy. (WFO ID: wfo-0000531669; IPNI LSID: urn:lsid:ipni.org:names:892748-1; accessed on 13 August 2026). For consistency with the previous report [17], the name *A. brevipedunculata* is used throughout this paper. A voucher specimen (KRIBB-KR2013-1) was deposited at the Functional Biomaterials Research Center. Dried and pulverized vines of *A. brevipedunculata* (9 kg) were extracted with 95% (*v*/*v*) ethanol at 22 ± 2 °C. The ethanol extract was concentrated under reduced pressure to 337.8 g crude extract. The crude extract was suspended in 1.5 L water and progressively partitioned with ethyl acetate. Concentration of the ethyl acetate-soluble fraction yielded 130.1 g of ABE. The dried extract was dissolved in dimethyl sulfoxide (DMSO; Sigma-Aldrich Corp., St. Louis, MO, USA) and 0.5% (*w*/*v*) carboxymethylcellulose (Sigma-Aldrich Corp) in saline prior to use in the in vitro and in vivo experiments, respectively.

#### 4.1.2. Chromatographic Isolation of Compound A–E

The isolation and structural identification of the compounds A–E were described in a previous report [17]. Their ^1^H, ^13^C NMR and ESI-MS spectra are provided in the Supplementary Materials (Figures S6–S20). Briefly, ABE was separated by silica gel column chromatography (40 × 10 cm, 200–400 mesh, Merck, Rahway, NJ, USA) into 38 subfractions (ABE1–ABE38) using a solvent system comprising a stepwise CHCl_3_/MeOH (1:0–0:1, *v*/*v*) gradient. ABE27 (37.6 g) was separated by silica gel column chromatography and eluted with CHCl_3_ and MeOH (1:0–0:1, *v*/*v*) to yield compound D (4.5 g). Compound A (4.3 mg) was isolated from ABE23A4 (283.4 mg) by preparative HPLC (C18; 15% (*v*/*v*) MeOH; 6 mL/min). Compound B (3.5 mg) was separated by silica gel column chromatography, eluted with CHCl_3_ and MeOH (1:0–0:1, *v*/*v*), and isolated by recrystallization of ABE32 (159.8 mg). Compound E (4.3 mg) was isolated from ABE19 (2.9 g) by C18 MPLC (130 g) using a 10–100% (*v*/*v*) MeOH gradient, followed by recrystallization. ABE23 (10.8 g) was fractionated by silica gel MPLC (120 g) using an *n*-hexane/EtOAc gradient (5:1–0:1, *v*/*v*) yielding 13 subfractions ABE23A–ABE23M. Subfraction 12 (ABE23L) afforded compound C (56.1 mg). The ABE fraction and compounds A–E used in the present study were obtained from the same batches prepared for our previous study [17].

### 4.2. Materials

Dinitrophenyl-human serum albumin (DNP-HSA), anti-DNP IgE, *o*-phthaldialdehyde, 4-nitrophenyl-*N*-acetyl-β-*D*-glucosaminide, and ovalbumin (OVA) were purchased from Sigma-Aldrich Corp. The alum adjuvant was acquired from Thermo Fisher Scientific (Waltham, MA, USA). Enzyme-linked immunosorbent assay (ELISA) kits for mouse cytokines (TNF-α, IL-1β, IL-4, IL-5, IL-6, and IL-10), antibodies (IgG1, IgG2a, and IgE) and rat cytokines (TNF-α, IL-4, and IL-6) were purchased from BD Biosciences (Franklin Lakes, NJ, USA). ELISA kits for myeloperoxidase (MPO) and eosinophil peroxidase (EPO) were obtained from R&D Systems (Minneapolis, MN, USA) and MyBiosource (San Diego, CA, USA), respectively. The corticosteroid dexamethasone (DEX; D4902; Sigma-Aldrich Corp., St. Louis, MO, USA) used in clinical asthma treatment served as a positive control [52]. The flavonoid quercetin (Q; Q4951; Sigma-Aldrich Corp) known for its anti-inflammatory and antioxidant properties served as a positive control [53].

### 4.3. Animals

Female BALB/c mice (age 6 weeks) were obtained from Orient Bio (Gwangju, Republic of Korea). Standard rodent chow and filtered water were provided *ad ibitum* throughout the study. The animals were maintained in a laminar airflow room maintained at 22 ± 2 °C, 55 ± 5% relative humidity, and a 12 h light/dark cycle. All mice were housed at a density of eight per cage.

### 4.4. OVA-Induced Allergic Airway Inflammation

Fifty-six mice were randomly divided into seven groups (*n* = 8 per group). The mice were assigned to the following experimental groups: phosphate-buffered saline (PBS) only (negative control); 300 mg/kg ABE only; OVA sensitization/challenge only; OVA sensitization/challenge plus 30 mg/kg, 100 mg/kg, or 300 mg/kg ABE; and 10 mg/kg DEX. To establish an eosinophilic airway inflammation model, the classical 21-day allergic asthma model was established [49]. Sensitization phase (Days 0 and 7): Mice were sensitized by intraperitoneal injection of OVA mixture (100 μg OVA and 4 mg alum adjuvant in 200 μL PBS). Challenge phase (Days 14, 15, 16 and 17): Mice were challenged with an aerosol of 3% (*w*/*v*) OVA solution (in PBS) for 30 min on days 14, 15 and 16. On day 17, the concentration of OVA solution was increased to 5% (*w*/*v*). The concentration of the OVA solution was determined from previous studies [54]. Aerosol challenges were administered using an ultrasonic nebulizer (NE-U17; OMRON Corp., Tokyo, Japan). ABE or reference drugs were orally administered once daily 1 h before the OVA challenge. Control mice were administered PBS by intraperitoneal injection on days 0 and 7 and then challenged with PBS on days 14, 15, 16 and 17. At 24 h after the final OVA challenge, mice were euthanized with CO_2_, and blood samples were obtained by cardiac puncture. Bronchoalveolar lavage fluid (BALF) was collected by performing three sequential washes with 0.5 mL of PBS through a tracheal cannula. The collected BALF was centrifuged at 400× *g*, 4 °C for 10 min. The supernatants were transferred to new microtubes and stored at −80 °C until analysis. The BALF cell pellets were resuspended in 100 μL PBS, mounted on slides, and stained with Diff-Quik staining solution (Sysmex Co., Kobe, Japan). Eosinophils, macrophages, and neutrophils were then counted with a hemocytometer (Marienfeld, Thuringia, Germany).

### 4.5. Histological Pathology

For histological analysis, the left lobes of the lung were fixed in a 4% paraformaldehyde solution for 72 h, dehydrated, and subsequently embedded in paraffin. The paraffin-embedded lung tissues were sectioned at a thickness of 3 μm and mounted onto slides. Hematoxylin and Eosin (H&E), Periodic acid-Schiff (PAS), Masson’s trichrome, and Toluidine blue staining were performed on the tissue sections. The pathological changes in the lung tissues were evaluated using a light microscope (at 200× magnification).

### 4.6. Cell Culture

RBL-2H3 (rat basophilic leukemia) cells were provided by the Department of Pharmacology, School of Medicine, Kyungpook National University. RBL-2H3 cells were cultured in Dulbecco’s modified Eagle’s medium (DMEM; Gibco, Grand Island, NY, USA) containing heat-inactivated 10% (*v*/*v*) fetal bovine serum (FBS; Gibco), 1% Antibiotic-Antimycotic (Gibco, Grand Island, NY, USA) at 37 °C in 5% CO_2_. RBL-2H3 cells at passages 4–8 were used throughout the study.

### 4.7. Cell Viability

Cell viability was measured using the MTT assay kit (WelGENE, Seoul, Republic of Korea). RBL-2H3 cells (3 × 10^4^/well in 96-well plates) were pretreated with various concentrations of ABE or its compounds for 24 h and subsequently incubated with 1 mg/mL of MTT reagent for 2 h at 37 °C. To dissolve the formed formazan crystals, 100 μL of DMSO was added to each well. The absorbance was measured at 570 nm in a spectrophotometer (Varioskan LUX multimode microplate reader; Thermo Fisher Scientific). Cell viability was calculated as a percentage relative to the control group.

### 4.8. β-Hexosaminidase Release

To evaluate mast cell degranulation, the release of β-hexosaminidase was measured. RBL-2H3 cells (5 × 10^5^/well in 12-well plates) sensitized with anti-DNP IgE (100 ng/mL) were washed 3 times with PBS, pretreated with ABE or its compounds for 1 h, and stimulated with 100 ng/mL DNP-HSA for 4 h at 37 °C. After incubation, the conditioned media were centrifuged at 150× *g* for 5 min at 4 °C and supernatants were transferred to a new microtube. To measure the intracellular amount of β-hexosaminidase, the cell pellets were lysed with 0.5% (*v*/*v*) Triton X-100 (Sigma-Aldrich Corp), and centrifuged at 150× *g* for 5 min at 4 °C. The 40 μL of supernatant and cell lysate was transferred to 96-well plates and mixed with an equal volume of substrate solution (1 mM 4-nitrophenyl-*N*-acetyl-β-*D*-glucosaminide in 0.1 M citrate buffer; pH 4.5) and then incubated at 37 °C for 1 h. To stop the reaction, 200 μL of 0.1 M Na_2_CO_3_-NaHCO_3_ solution (pH 10) was added. In the case of BALF, β-hexosaminidase levels were measured using only the supernatant. Absorbance was measured at 405 nm using a spectrophotometer (Thermo Fisher Scientific). β-hexosaminidase release (%) was calculated relative to the total enzyme activity.

β-hexosaminidase release (%) = OD cultured media/(OD cultured media + OD cell lysate) × 100

### 4.9. Histamine Release

To assess mast cell degranulation, the histamine levels in the conditioned media were quantified. RBL-2H3 cells (5 × 10^5^/well in 12-well plates) sensitized with anti-DNP IgE (100 ng/mL) were washed 3 times with PBS, pretreated with ABE or compounds of ABE for 1 h, and stimulated with 100 ng/mL DNP-HSA for 4 h at 37 °C. The conditioned media were collected, centrifuged at 150× *g* for 5 min at 4 °C and then the supernatants were transferred into new microtubes. For histamine extraction, 80 μL of 0.1 N HCl and 20 μL of 60% (*v*/*v*) perchloric acid were added to the 200 μL of conditioned media, vortexed, and centrifuged at 18,000× *g* for 20 min at 4 °C. The 200 μL of supernatant was transferred to a 1.5 mL microtube, and vortexed with 200 μL of 5 M NaCl, 100 μL of 5 N NaOH, and 800 μL of *n*-butanol, and centrifuged at 18,000× *g* for 20 min at 4 °C. The 500 μL of supernatant was transferred to a 1.5 mL microtube, and vortexed with 200 μL of 0.1 N HCl and 500 μL of *n*-heptane and centrifuged at 18,000× *g* for 20 min at 4 °C. To quantify the concentration of released histamine, the extracted aqueous phase was reacted with *o*-phthaldialdehyde solution (in MeOH). Fluorescent intensity was measured at an excitation wavelength of 380 nm and an emission wavelength of 440 nm using a fluorescent plate reader (Molecular Devices LLC, Sunnyvale, CA, USA).

### 4.10. Intracellular Calcium

To measure intracellular calcium levels, the fluorescence indicator Fluo-3/AM (Invitrogen, Carlsbad, CA, USA) was used. RBL-2H3 cells (2 × 10^4^/well in 96-well plates) sensitized with anti-DNP IgE (100 ng/mL) were pre-incubated with 5 μM Fluo-3/AM at 37 °C for 1 h. The cells were washed 3 times with Hank’s Balanced Salt Solution (HBSS; Thermo Fisher Scientific), pretreated with ABE or its compounds for 1 h, and then stimulated with 100 ng/mL DNP-HSA. BAPTA-AM (Thermo Fisher Scientific), a calcium chelator, was used as a positive control. Fluorescent intensity was measured using a fluorescent plate reader (Molecular Devices LLC) at an excitation wavelength of 485 nm and an emission wavelength of 510 nm. The intracellular calcium levels were normalized relative to the untreated control.

### 4.11. Quantitative Polymerase Chain Reaction (qPCR)

To evaluate the mRNA expression levels of inflammatory cytokines, qPCR was performed using lung tissues from an OVA-induced airway inflammation mouse model and the DNP-HSA-challenged RBL-2H3 cells. The right superior lobe of the mouse lung tissue was used for RNA extraction. For RNA extraction, 5 mg of lung tissue was minced into small pieces. RBL-2H3 cells (5 × 10^5^/well in 12-well plates) sensitized with anti-DNP IgE (100 ng/mL) were pretreated with ABE or its compounds for 1 h and then stimulated with 100 ng/mL DNP-HSA for 1 h at 37 °C. Total RNA was isolated using an Ambion RNA isolation kit (Thermo Fisher Scientific) according to the manufacturer’s protocol. The isolated RNA was quantified using a NanoDrop spectrophotometer (Thermo Fisher Scientific, Waltham, MA, USA), 1 μg of total RNA was reverse transcribed into cDNA using a cDNA synthesis kit (Thermo Fisher Scientific) on a Bio-Rad T100 thermal cycler (Bio-Rad Laboratories, Hercules, CA, USA). The qPCR was conducted using a CFX96 Touch Deep Well Real-Time PCR Detection System (Bio-Rad Laboratories) according to the manufacturer’s protocol. Briefly, 1.5 μL cDNA (150 ng), 1 μL of each forward and reverse primer (0.4 µM), 12.5 μL SYBR Premix Ex Taq (Takara Bio Inc., Shiga, Japan), and 9 μL dH_2_O were combined in a reaction tube to a total volume of 25 μL. The amplification conditions for the PCR were similar to those previously reported (Denaturation step: 10 s at 95 °C, Amplification step: 40 cycles of 5 s at 95 °C and 30 s at 60 °C, Melting curve analysis: 15 s at 95 °C, 30 s at 60 °C, and 15 s at 95 °C [32]). Relative quantification of mRNA expression was determined with Precision Melt Analysis Software version 1.3 (Bio-Rad Laboratories). The primer sequences are listed in Table S1. The cycle number (40 cycles) was optimized to ensure exponential product accumulation. β-actin was the endogenous control for gene expression ratio normalization.

### 4.12. Enzyme-Linked Immunosorbent Assay (ELISA)

To measure the levels of cytokines (TNF-α, IL-1β, IL-4, IL-5, IL-6, and IL-10) and enzymes (MPO and EPO) in BALF, Igs (IgG1, IgG2a, and IgE) in serum, and cytokines (TNF-α, IL-4 and IL-6) in conditioned media, ELISA kits were used according to the manufacturer’s protocol. To measure OVA-specific IgE, an immune plate was coated with 20 μg OVA instead of antibody. To analyze cytokines such as TNF-α, IL-4, and IL-6, RBL-2H3 cells (5 × 10^5^/well in 12-well plates) sensitized with anti-DNP IgE (100 ng/mL) were washed 3 times with PBS, pretreated with ABE for 1 h, and stimulated with 100 ng/mL DNP-HSA for 6 h at 37 °C. The assay was performed in a 96-well Nunc-immune plate (Thermo Fisher Scientific). Optical density was measured using a spectrophotometer (Thermo Fisher Scientific) at 450 nm within 10 min after adding the stop solution (2N H_2_SO_4_).

To analyze phosphorylation of signaling molecules from DNP-HSA-challenged RBL-2H3 cell lysate, an ELISA kit (p-p38; #7946CA; Cell Signaling Technology, Danvers, MA, USA) or specific monoclonal antibodies (p-ERK, #7246S; p-JNK, #7141S; p-IκBα, #7343S; p-NF-κB, #7834S; Cell Signaling Technology) were used according to the manufacturer’s protocol. RBL-2H3 cells (10^6^/well in 6-well plates) sensitized with anti-DNP IgE (100 ng/mL) were washed 3 times with PBS, pretreated with ABE or its compounds for 1 h, and stimulated with 100 ng/mL DNP-HSA for 30 min at 37 °C to evaluate ERK, JNK, and p38 phosphorylation or for 1 h to evaluate IκBα and NF-κB phosphorylation. Then, activated cells were washed with PBS and lysed with cell lysis buffer (Cell Signaling Technology) containing 0.5 mM PMSF/DTT and protease-phosphatase inhibitor solution (Thermo Fisher Scientific). The cell lysate samples were vortexed, incubated on ice for 30 min, and centrifuged at 400× *g* for 30 min at 4 °C. The supernatants were collected and protein concentrations were determined using a Bradford protein assay (Bio-Rad Laboratories) according to the manufacturer’s protocol.

### 4.13. Western Blot

Western blotting was performed to evaluate the phosphorylation levels of signaling proteins in lysates from DNP-HSA-stimulated RBL-2H3 cells. RBL-2H3 cells (10^6^/well in 6-well plates) sensitized with anti-DNP IgE (100 ng/mL) were washed 3 times with PBS, pretreated with ABE or its compounds for 1 h, and stimulated with 100 ng/mL DNP-HSA for 7 min to detect Lyn and Syk and for 30 min to detect PI3K, PLCγ, and Akt. Total protein was extracted as previously described in Section 4.12. Equal amounts of protein were mixed with loading buffer, boiled at 95 °C for 5 min, separated on 8–12% SDS-PAGE gels and transferred onto nitrocellulose membranes (Bio-Rad Laboratories). After blocking with 5% (*w*/*v*) bovine serum albumin (BSA) for 1 h at room temperature (R.T), the membranes were incubated with primary antibodies against target proteins overnight at 4 °C. The membranes were washed 3 times for 10 min each with Tris-buffered saline containing 0.1% Tween-20 (TBST). Then, membranes were incubated with anti-IgG horseradish peroxidase-conjugated secondary antibody (#1706515; Bio-Rad Laboratories; 1:3000) for 1 h at R.T. After washing 3 times with TBST, protein bands were visualized with a chemiluminescent substrate (Thermo Fisher Scientific). Chemiluminescent signals were detected using a ChemiDoc MP imaging system (Bio-Rad Laboratories). The signal intensities of the bands were quantified using ImageJ software version 1.54p (National Institutes of Health, Bethesda, MD, USA). The following antibodies were purchased from Cell Signaling Technology: phospho-Lyn (#2731; Tyr507, rabbit polyclonal, 1:1000); phospho-Syk (#2711; Tyr525/526, rabbit polyclonal, 1:1000); phospho-phosphoinositide 3-kinase (PI3K, #4228S; rabbit polyclonal, 1:1000); phospho-phospholipase C (PLCγ, #2821; Tyr783, rabbit monoclonal, 1:1000); phospho-Akt (#9271; Ser473, rabbit polyclonal, 1:1000); Lyn (#2732; rabbit polyclonal, 1:1000); Syk (#2712; rabbit polyclonal, 1:1000); PI3K (#4292S; rabbit polyclonal, 1:1000); PLCγ (#2822; rabbit monoclonal, 1:1000); Akt (#9272; rabbit polyclonal, 1:1000); and actin (#4967S; rabbit monoclonal, 1:1000).

### 4.14. Statistical Analysis

Data were analyzed in GraphPad Prism version 7 (GraphPad Software, La Jolla, CA, USA). Treatment effects were analyzed by one-way ANOVA followed by Dunnett’s test. *p* < 0.05 indicates a significant difference between treatment means.

## 5. Conclusions

In conclusion, we investigated the therapeutic potential of ABE against mast cell-mediated allergic airway inflammation. ABE suppressed airway hyperresponsiveness, innate immune cell activation, and inflammatory responses. The anti-allergic inflammatory effect of ABE was mediated by inhibition of mast cell activation through suppression of the calcium and inflammatory signaling pathways. Furthermore, the compounds isolated from ABE suppressed mast cell activation by inhibiting the signaling pathway. Taken together, our results suggest that ABE is a candidate for the alternative treatment of allergic airway inflammatory diseases.

## Data Availability

The data supporting the findings of this study are available from the corresponding author upon reasonable request.

## Supplementary Materials

The following supporting information can be downloaded at: https://www.mdpi.com/article/10.3390/molecules31193479/s1, Table S1: The primer sequences for the qPCR; Table S2: The isolated seventeen compounds from ABE; Figure S1: Effects of ABE on Muc5ac expression in lung tissue; Figure S2: Effects of ABE on FcεR1a expression in lung tissue; Figure S3: Effects of ABE on translocation of transcription factors; Figure S4: The inhibitory effect of isolated compounds of ABE; Figure S5: Structures of five effective compounds A–E; Figure S6: ESIMS spectrum of syringic acid (compound A); Figure S7: ^1^H NMR (600 MHz, methanol-*d*_4_) spectrum of syringic acid (compound A). Figure S8: ^13^C NMR (150 MHz, methanol-*d*_4_) spectrum of syringic acid (compound A); Figure S9: ESIMS spectrum of ellagic acid (compound B); Figure S10: ^1^H NMR (600 MHz, DMSO-*d*_6_) spectrum of ellagic acid (compound B); Figure S11: ^13^C NMR (100 MHz, DMSO-*d*_6_) spectrum of ellagic acid (compound B); Figure S12: ESIMS spectrum of resveratrol (compound C); Figure S13: ^1^H NMR (400 MHz, methanol-*d*_4_) spectrum of resveratrol (compound C); Figure S14: ^13^C NMR (100 MHz, methanol-*d*_4_) spectrum of resveratrol (compound C); Figure S15: ESIMS spectrum of catechin (compound D); Figure S16: ^1^H NMR (400 MHz, methanol-*d*_4_) spectrum of catechin (compound D); Figure S17: ^13^C NMR (100 MHz, methanol-*d*_4_) spectrum of catechin (compound D); Figure S18: ESIMS spectrum of 3,3′4′-O-tri-methylellagic acid 4-*O*-*β*-D-glucopyranoside (compound E); Figure S19: ^1^H NMR (600 MHz, DMSO-*d*_6_) spectrum of 3,3′4′-O-tri-methylellagic acid 4-*O*-*β*-D-glucopyranoside (compound E); Figure S20: ^13^C NMR (150 MHz, DMSO-*d*_6_) spectrum of 3,3′4′-O-tri-methylellagic acid 4-*O*-*β*-D-glucopyranoside (compound E).

## Abbreviations

ABE, ethyl acetate fraction of *Ampelopsis brevipedunculata*; OVA, ovalbumin; BALF, bronchoalveolar lavage fluid; Th2 cells, T-helper type 2 cells; APCs, antigen-presenting cells; Dex, dexamethasone; DMSO, dimethyl sulfoxide; DNP-HSA, Dinitrophenyl-human serum albumin; ELISA, Enzyme-linked immunosorbent assay; TNF, tumor necrosis factor; IL, Interleukin; PBS, phosphate-buffered saline; H&E, Hematoxylin and Eosin; PAS, Periodic acid-Schiff; MPO, myeloperoxidase; EPO, eosinophil peroxidase; ROS, Reactive oxygen species.
