# Supplementary material for: Effects of Ampelopsis brevipedunculata on Ovalbumin-Induced Allergic Airway Inflammation via Regulating Mast Cell Activation and PLCγ/Akt/NF-κB Signaling Pathway
Source: Molecules. 2026 Sep 29;31(19):3479. doi: 10.3390/molecules31193479 (PMC13666435; doi:10.3390/molecules31193479)
Supplement: Supplementary file 1 [file molecules-31-03479-s001.zip › molecules-4510226-supplementary.pdf]

Supplementary information

Effects of *Ampelopsis brevipedunculata* on Ovalbumin-Induced Allergic Airway Inflammation via Regulating Mast Cell Activation and PLCγ/Akt/NF-κB Signaling Pathway

Yeon-Yong Kim [1,†], Hyun-Jae Jang [1,†], Seungwon Jeong [2,3,†], Seung Woong Lee [1], Sang-Hyun Kim [4,*] and Soyoung Lee [3,*]

[1] Functional Biomaterial Research Center, Korea Research Institute of Bioscience and Biotechnology (KRIBB), 181, Ipsin-gil, Jeongeup 56212, Republic of Korea; saxyyou12@gmail.com (Y.-Y.K.); water815@kribb.re.kr (H.-J.J.); lswdoc@kribb.re.kr (S.W.L.)

[2] Department of Bionanotechnology and Bioconvergence Engineering, Jeonbuk National University, 567, Baekje-daero, Jeonju 54896, Republic of Korea; uka167@jbnu.ac.kr

[3] Department of Innovative Pharmaceutical Sciences, Advanced Institute of Science and Technology, Kyungpook National University, 80, Daehak-ro, Buk-gu, Daegu 41566, Republic of Korea

[4] Cell Matrix Research Institute, Department of Pharmacology, School of Medicine, Kyungpook National University, 680, Gukchaebosang-ro, Jung-gu, Daegu 41944, Republic of Korea

* Correspondence: shkim72@knu.ac.kr (S.-H.K.); sy.lee@knu.ac.kr (S.L.); Tel.: +82-53-420-4838 (S.-H.K.); +82-53-950-4540 (S.L.)

† These authors contributed equally to this work.

1. Materials and Methods

1.1 Western blot

Western blotting was performed to evaluate the translocation of transcription factors in nucleus lysates form DNP-HSA-stimulated RBL-2H3 cells. RBL-2H3 cells (10^6/well in 6-well plates) sensitized with anti-DNP IgE (100 ng/mL) were washed 3 times with PBS, pretreated with ABE or its compounds for 1 h, and stimulated with 100 ng/mL DNP-HSA for 1 h to detect NFAT1 and NF-κB. Nucleus protein was extracted using NE-PER™ Nuclear and Cytoplasmic Extraction Reagents (Thermo Fisher Scientific) according to the manufacturer's protocol. The nucleus lysates were collected, and protein concentrations were determined using a Bradford protein assay (Bio-Rad) according to the manufacturer's protocol. 10 μg of nucleus protein were mixed with loading buffer, boiled at 95 °C for 5 min, separated on Nupage™ 4 ~ 12%, Bis-Tris, 1.5 mm, Mini Protein Gels (Invitrogen) and transferred onto polyvinyl fluoride (PVDF) membrane pre-activated with methanol for 5 min. After blocking with 5% (w/v) bovine serum albumin (BSA) for 1h at room temperature (R.T), the membranes were incubated with primary antibodies against target proteins over-night at 4 °C. The membranes were washed 3 times for 10 min each with Tris-buffered saline containing 0.1 % Tween-20 (TBST). Then, membranes were incubated with anti-IgG horseradish peroxidase-conjugated secondary antibody for 1h at R.T. After washing 3 times with TBST, protein bands were visualized with a chemiluminescent substrate (Thermo Fisher Scientific). Chemiluminescent signals were detected using a Chemi-Doc MP imaging System (Bio-Rad). The signal intensities of the bands were quantified using ImageJ software (National Institutes of Health, Bethesda, MD, USA). The following antibodies were purchased from Cell Signaling Technology, Santa Cruze Biotechnology (Dallas, TX, USA) and Bio-Rad: NFAT1 (#5862; rabbit polyclonal, 1:1,000); NF-κB p65 (#8242; rabbit polyclonal, 1:1,000); Lamin B1 (#sc-374015; mouse monoclonal, 1:1,000), Goat Anti-Rabbit IgG (H + L)-HRP Conjugate (#1706515; 1:2000), Goat Anti-Mouse IgG (H + L)-HRP Conjugate (#1706516; 1:2000).

1.2 Immunohistochemistry (IHC)

Immunohistochemistry was performed to evaluate the expression of Muc5ac and FcεR1α. The left lobes of lung were fixed in 4% paraformaldehyde solution for 72 h, dehydrated, and subsequently embedded in paraffin. The paraffin-embedded lung tissues were sectioned at a thickness of 3 μm and mounted onto slides. Paraffin-embedded lung tissue sections were deparaffinized in oven at 60 °C for 1h. Sections were then immersed in xylene 3 times for 5 min each to remove residual paraffin. Tissue rehydration was performed using a graded ethanol series

(100%, 95%, 80%, 70% and 50%) for 5 min each. Antigen retrieval was performed in citrate buffer (pH 6.0) using a microwave for 18 min. Endogenous peroxidase activity was blocked with 3% hydrogen peroxide (H_2O_2) in methanol for 10 min. Sections were then washed with PBST (0.05% Tween 20) 3 times for 5 min. Nonspecific binding was blocked with blocking buffer containing 3% bovine serum albumin (BSA) in PBST for 30 min at R.T. Sections were then incubated overnight at 4 °C with primary antibodies against Muc5ac and FcεR1α, diluted in PBST containing 1% BSA. After washing with PBST 3 times for 5 min each, sections were incubated with a biotinylated secondary antibody for 30 min at room temperature. After washing with PBST 3 times for 5 min each, sections were incubated with avidin-biotin-peroxidase complex (Vectastain ABC kit, Vector Laboratories, Burlingame, CA, USA) for 30 min at R.T. Immunoreactivity was visualized using a 3,3′-diaminobenzidine (DAB) substrate, and the reaction was terminated by rinsing with distilled water. Sections were then counterstained with Mayer's hematoxylin, followed by treatment with bluing reagent. After staining, sections were mounted using an aqueous mounting medium. The representative images were captured using a light microscope (100× or 200× magnification) Muc5ac (#61192; rabbit monoclonal, 1:250); FcεR1α (16992; rabbit monoclonal, 1:200), Goat Anti-Rabbit IgG Antibody (H+L), Biotinylated, R.T.U. (BP-9100-50, 1:200).

1.3 Histamine release

To assess mast cell degranulation, the histamine levels in the conditioned media were quantified. RBL-2H3 cells (5×10^5/well in 12-well plates) sensitized with anti-DNP IgE (100 ng/mL) were washed 3 times with PBS, pretreated with ABE or compounds of ABE for 1 h, and stimulated with 100 ng/mL DNP-HSA for 4 h at 37 °C. The conditioned media were collected, centrifuged at 150 ×*g* for 5 min at 4 °C and then supernatants were transferred into new microtubes. For histamine extraction, 80 μL of 0.1 N HCl and 20 μL of 60% (v/v) perchloric acid were added with the 200 μL of conditioned media, vortexed, and centrifuged at 18,000 ×*g* for 20 min at 4 °C. The 200 μL of supernatant was transferred to a 1.5 mL microtubes, and vortexed with 200 μL of 5 M NaCl, 100 μL of 5 N NaOH, and 800 μL of *n*-butanol, and centrifuged at 18,000 ×*g* for 20 min at 4 °C. The 500 μL of supernatant was transferred to a 1.5-mL microtube, and vortexed with 200 μL of 0.1 N HCl and 500 μL of *n*-heptane and centrifuged at 18,000 ×*g* for 20 min at 4 °C. To quantify the concentration of released histamine, the extracted aqueous phase was reacted with *o*-phthaldialdehyde solution (in MeOH). Fluorescent intensity was measured at excitation wavelength of 380 nm and emission wavelength of 440 nm using a fluorescent plate reader (Molecular Devices LLC, Sunnyvale, CA, USA)

Table S1. The primer sequences for the qPCR

Gene	Origin	Forward (5'- to -3')	Reverse (5'- to -3')	GenBank No.
TNF-α	Mouse	AAGCCTGTAGCCCACGTCGTA	GGCACCACTAGTTGGTTGTCTTTG	NM_013693
IL-1β	Mouse	ATAACCTGCTGGTGTGTGAC	AGGTGCTGATGTACCAGTTG	XM_006498795
IL-4	Mouse	TCTCGAATGTACCAGGAGCCATATC	AGCACCTTGGAAGCCCTACAGA	NM_021283
IL-5	Mouse	ATGGAGATTCCCATGAGCAC	AGCCCCTGAAAGATTTCTCC	NM_010558
IL-6	Mouse	CCACTTCACAAGTCGGAGGCTTA	GCAAGTGCATCATCGTTGTTCATAC	NM_001314054
IL-13	Mouse	CCTCTGACCCTTAAGGAGCTT	ATGTTGGTCAGGGAATCCAG	NM_008355
IL-17	Mouse	TCCCTCTGTCATCTGGGAAG	CTCGACCCTGAAAGTGAAGG	NM_010552
IL-33	Mouse	TCCAACTCCAAGATTTCCCCG	CATGCAGTAGACATGGCAGAA	XM_036161730
β-actin	Mouse	TAGACTTCGAGCAGGAGATG	TTGATCTTCATGGTGCTAGG	NM_007393
TNF-α	Rat	TCCCAAATGGGCTCCCTCTC	AAATGGCAAACCGGCTGACG	NM_012675
IL-1β	Rat	GCTGTGGCAGCTACCTATGTCTTG	AGGTCGTCATCATCCCACGAG	NM_031512
IL-4	Rat	ACCTTGCTGTCACCCTGTC	TTGTGAGCGTGGACTCATTC	NM_201270
IL-5	Rat	AGCTCTGTTGACGAGCAATGA	CCCTCGGACAGTTTGATTCTT	NM_021834
IL-6	Rat	TGTGCAATGGCAATTCTGAT	GAGCATTGGAAGTTGGGGTA	NM_012589
IL-13	Rat	CCACAGGACCCAGAGGATATTGA	TAGCGGAAAAGTTGCTTGGAGTAA	NM_053828
β-actin	Rat	GAAGCTGTGCTATGTTGCCCTAGA	GTACTCCTGCTTGCTGATCCACAT	NM_031144

Table S2. The isolated seventeen compounds from ABE

No.	Name
**1**	Betulin
**2**	Betulinic acid
**3**	β-Sitosterol
**4**	β-Sitosterol glucoside
**5**	Aromadendrinc
**6**	3-O-Glycoside-aromadendrin
**7**	Catechin
**8**	Gallic acid
**9**	Vanillic acid
**10**	Ethyl gallate
**11**	Ethyl gallyl 4-O-α-D-glucopyranoside
**12**	Syringic acid
**13**	Benzyl 6′-O-galloyl-β-D-glucopyranoside
**14**	Ellagic acid
**15**	3′-O-Methylellagic acid 4-O-α-L-rhamnopyranoside
**16**	4′-O-(β-D-Glucopyranosyl)-3,3′4-tri-O-methylellagic acid
**17**	Resveratrol

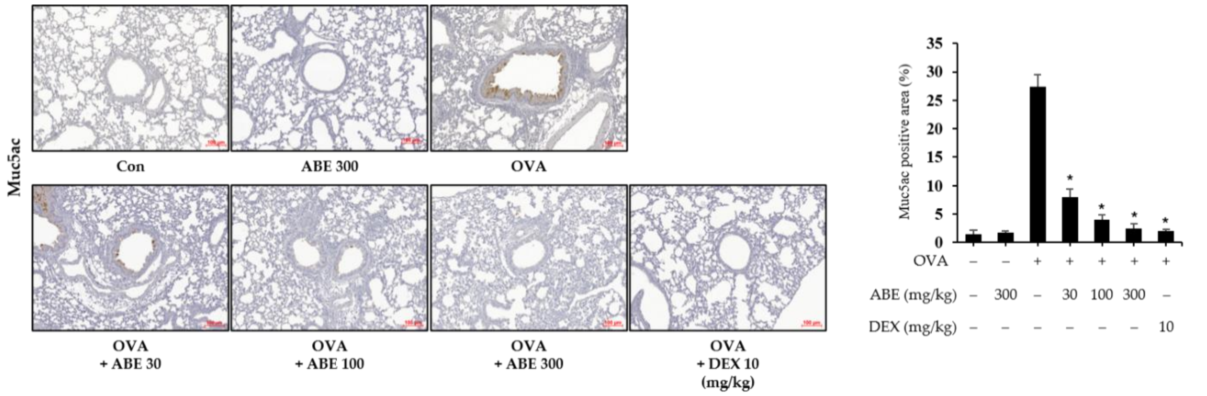

Figure S1. Effects of ABE on Muc5ac expression in lung tissue. IHC staining for Muc5ac was performed on the left lobes of the lung. The representative images were obtained at 100× magnification (Scale bar size: 100 μm). Data point represents the mean ± SEM (n=3 per group). Statistical significance was analyzed using one-way ANOVA followed by Dunnett's test. *$p < 0.05$. relative to OVA group

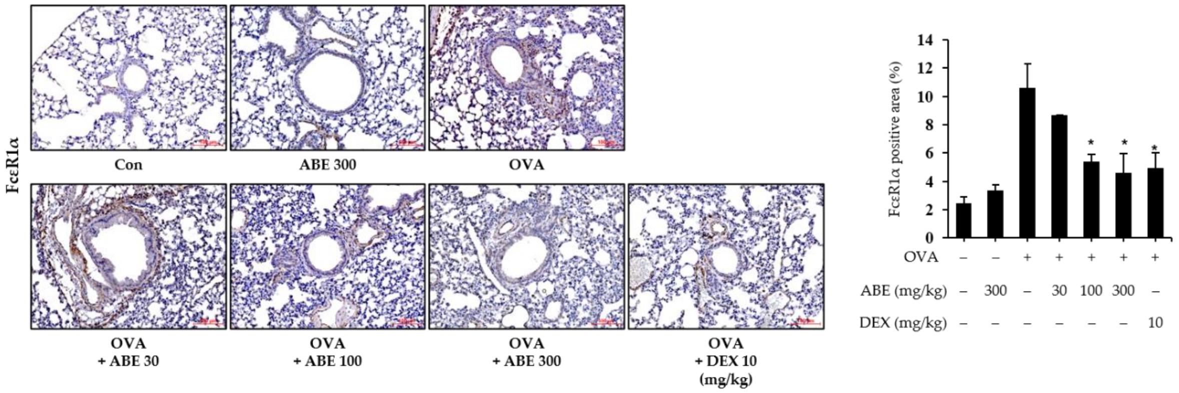

Figure S2. Effects of ABE on FcεR1a expression in lung tissue. IHC staining for FcεR1a was performed on the left lobes of the lung. The representative images were obtained at 200×magnification (Scale bar size: 100 μm). Data point represents the mean ± SEM (n=3 per group). Statistical significance was analyzed using one-way ANOVA followed by Dunnett's test. *p < 0.05. relative to OVA group

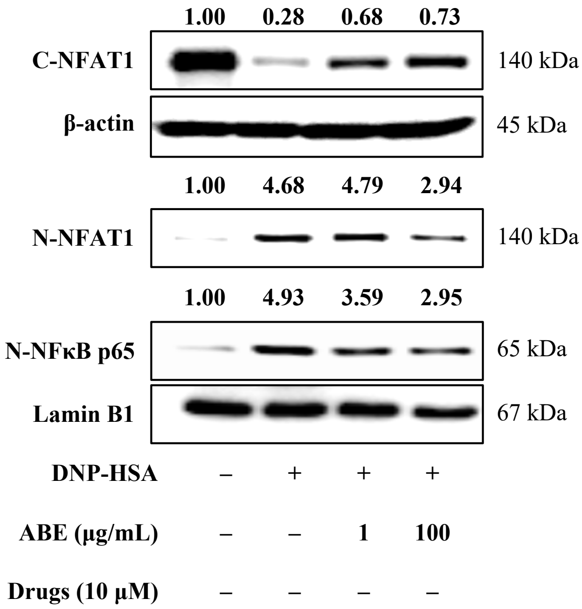

Figure S3. Effects of ABE on translocation of transcription factors. Protein samples were extracted from the nuclear and cytosol fraction of RBL-2H3 cells. NFAT1 and NF-κB were analyzed by western blotting. β-actin and Lamin B1 was used as an endogenous control for the nuclear fraction.

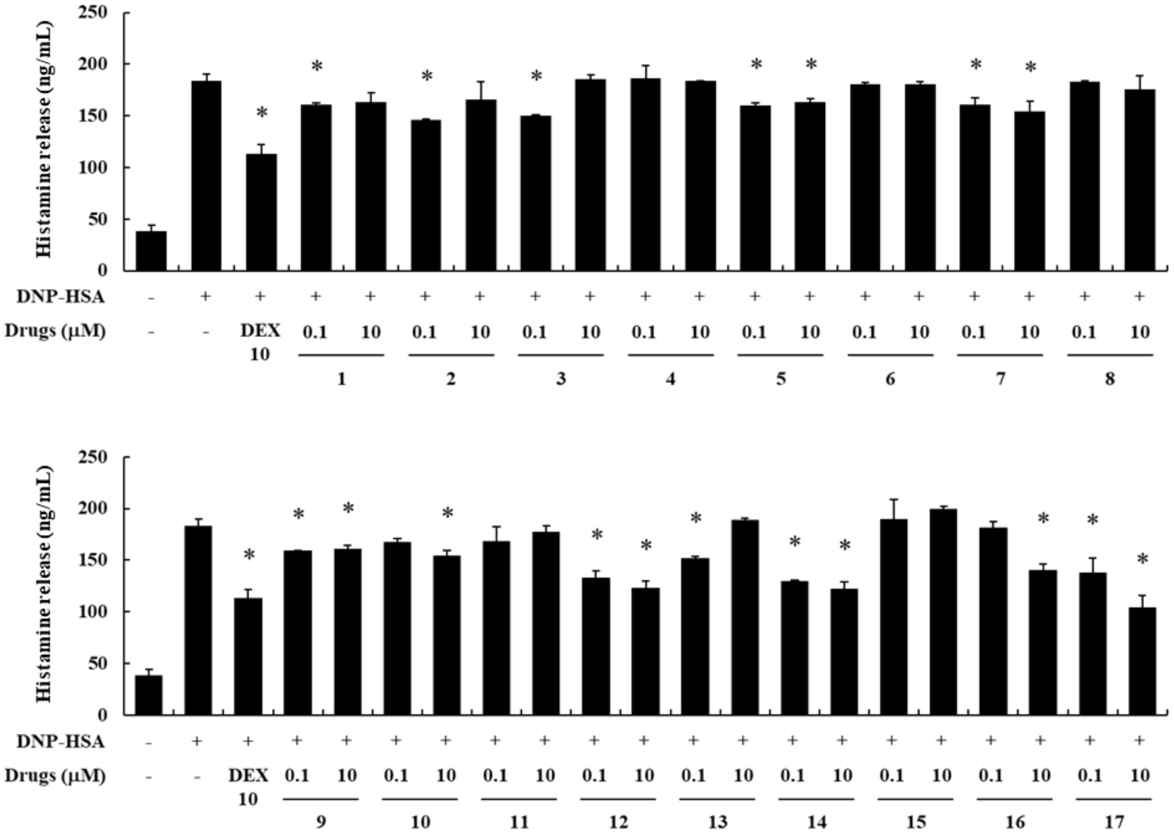

No.	12	14	17	7	16
**Compound**	Syringic acid	Ellagic acid	Resveratrol	Catechin	4′-O-(β-D-Glucopyranosyl)-3,3′4-tri-O-methylellagic acid
**Symbol**	**A**	**B**	**C**	**D**	**E**

Figure S4. The inhibitory effect of isolated compounds of ABE. The anti-allergic effect of isolated compounds was analyzed by mast cell degranulation assay on RBL-2H3 cells. DEX was used for effective positive control, and compounds were selected for similar efficacy compared to DEX. Data point represents the mean ± SEM (n=3 per group). Statistical significance was analyzed using one-way ANOVA followed by Dunnett's test. *$p < 0.05$. relative to DNP-HSA group

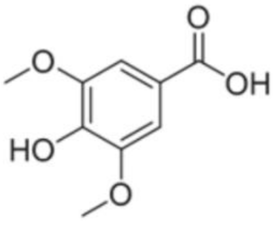

A, Syringic acid

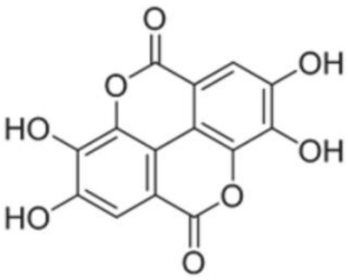

B, Ellagic acid

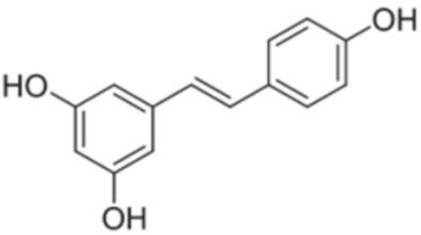

C, Resveratrol

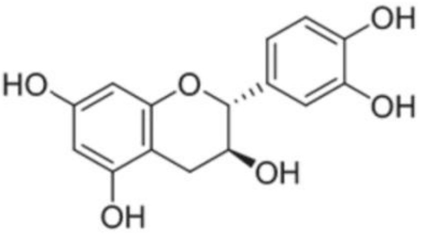

D, Catechin

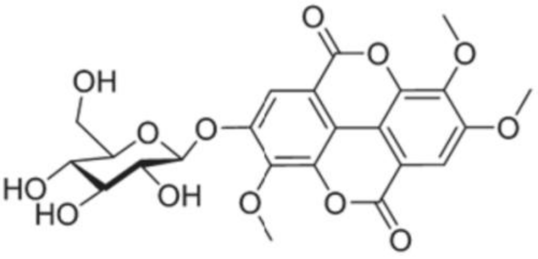

E, 4′-O-(β-D-Glucopyranosyl)-3,3′4-tri-O-methylellagic acid

Figure S5. Structures of five effective compounds A–E. The structures of the selected compounds that were analyzed for their inhibitory effect on mast cell degranulation.

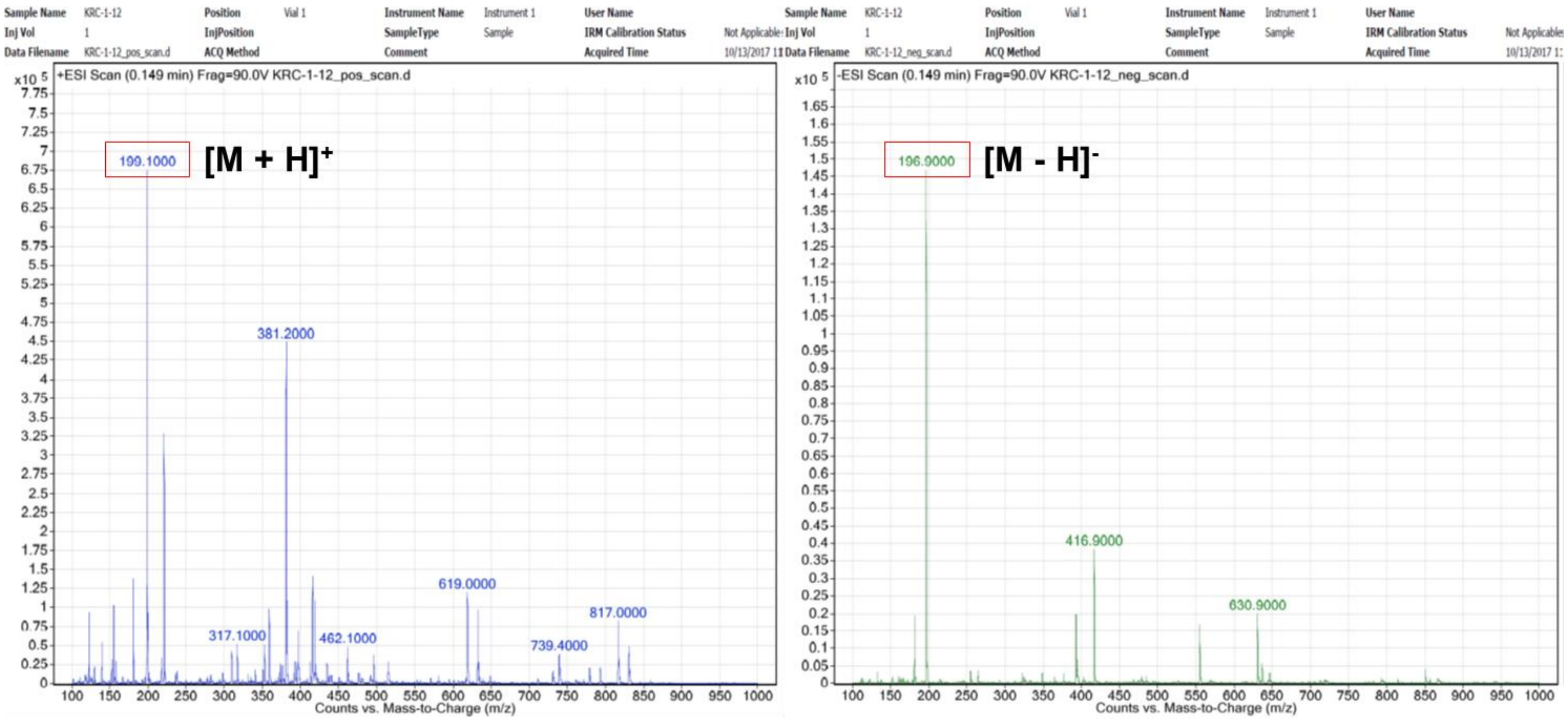

Figure S6. ESIMS spectrum of syringic acid (**compound A**)

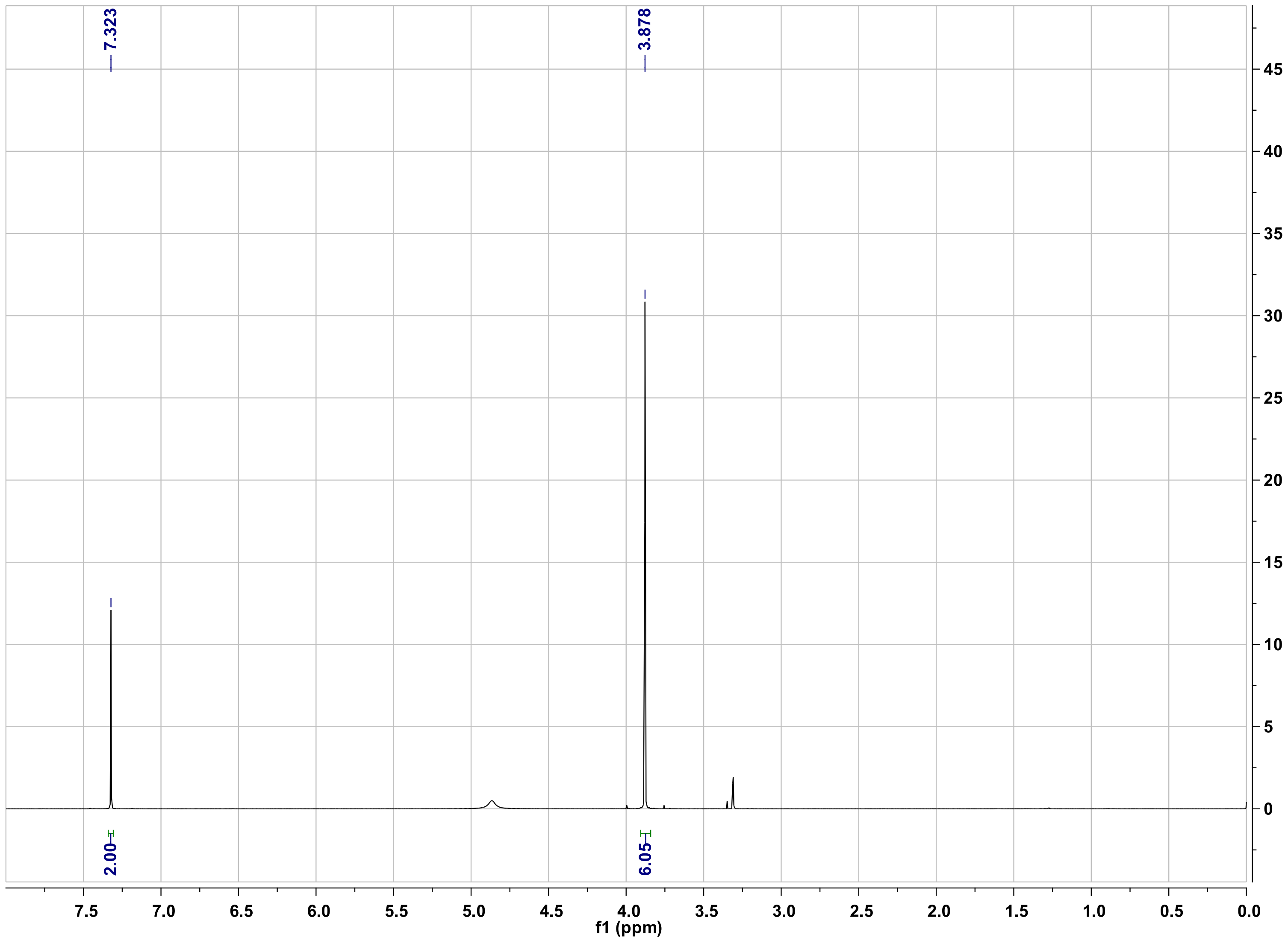

Figure S7. ^{1}H NMR (600 MHz, methanol-d_4) spectrum of syringic acid (**compound A**).

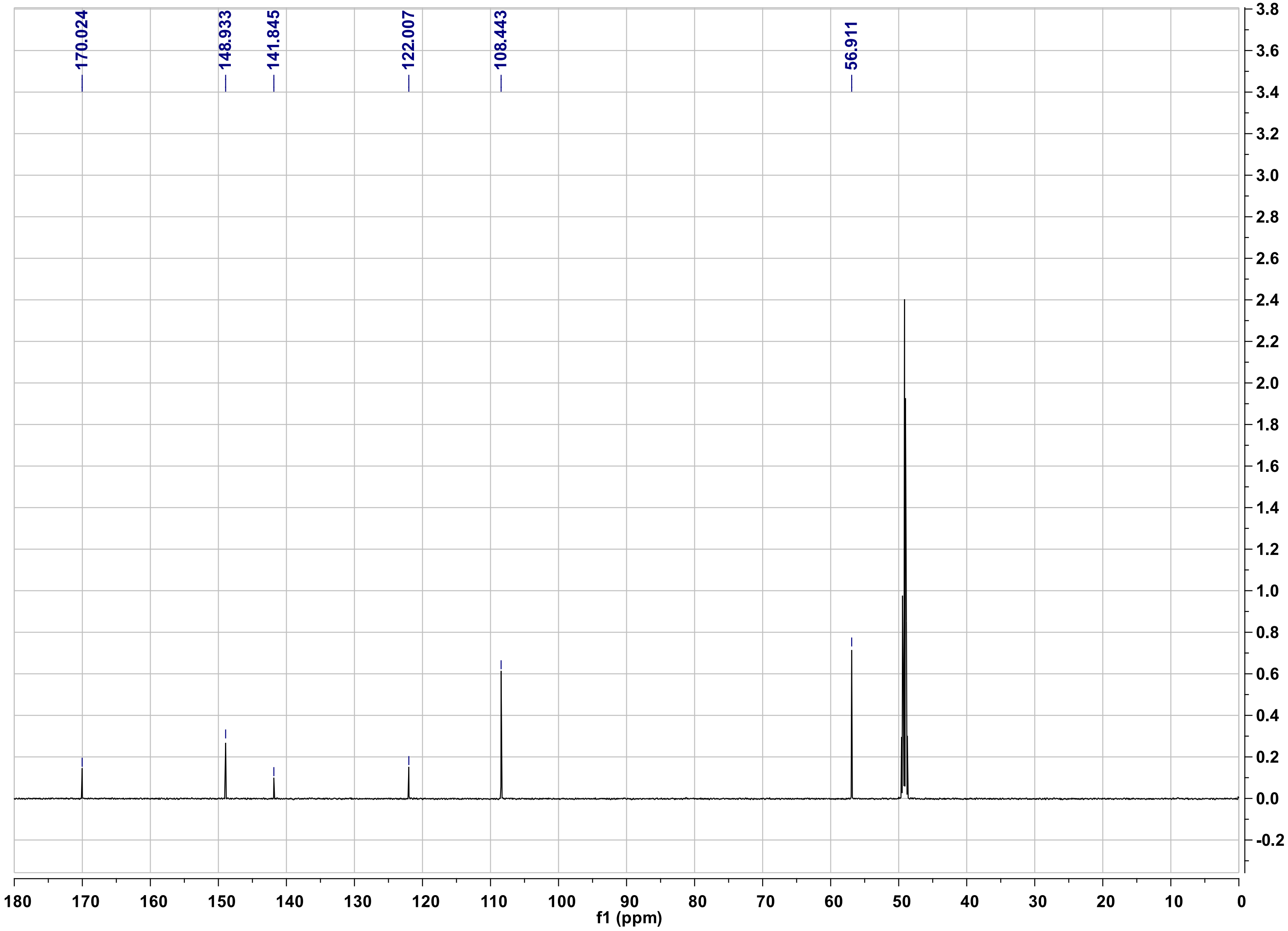

Figure S8. ^{13}C NMR (150 MHz, methanol-d_4) spectrum of syringic acid (**compound A**)

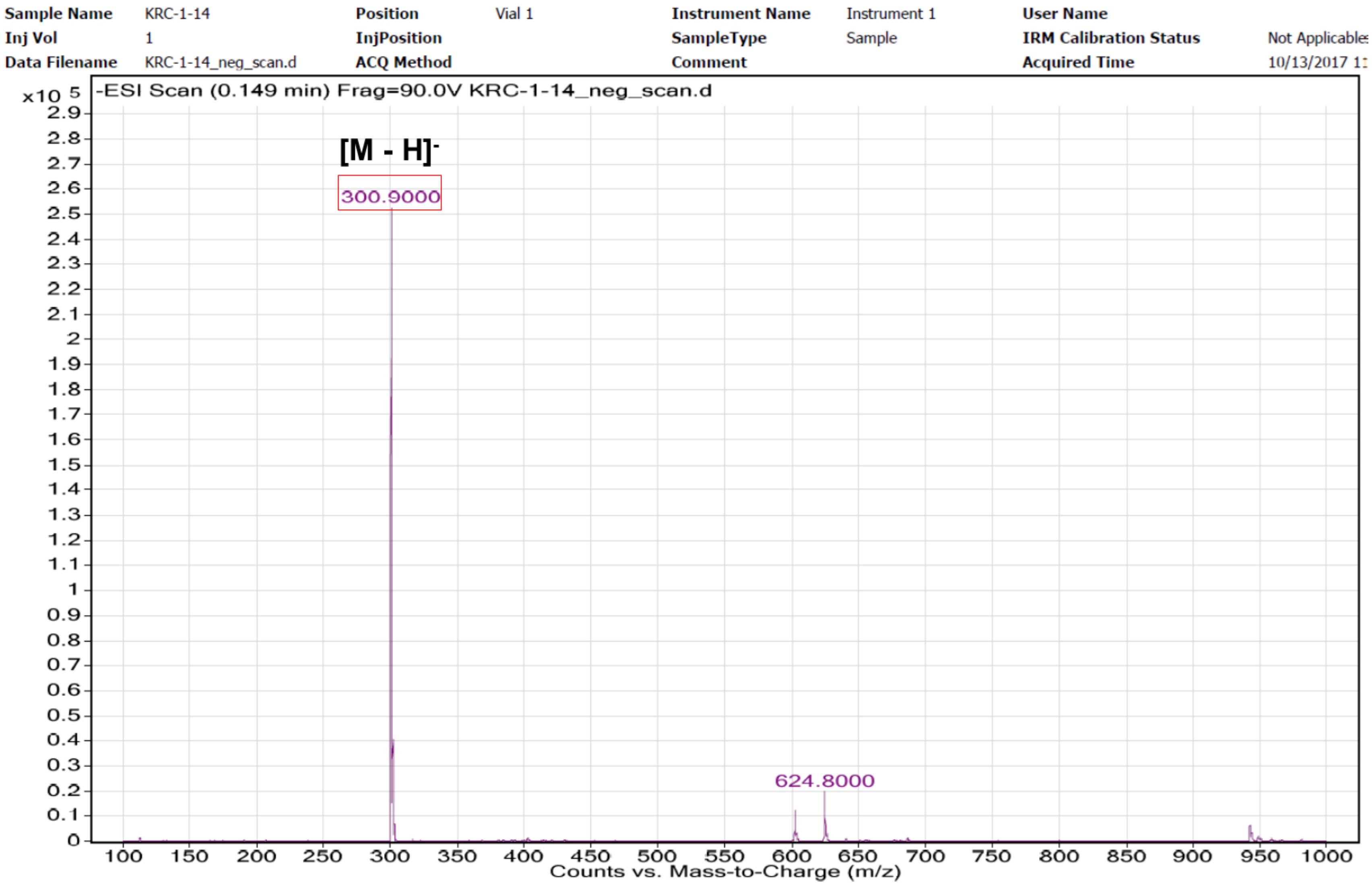

Figure S9. ESIMS spectrum of ellagic acid (**compound B**)

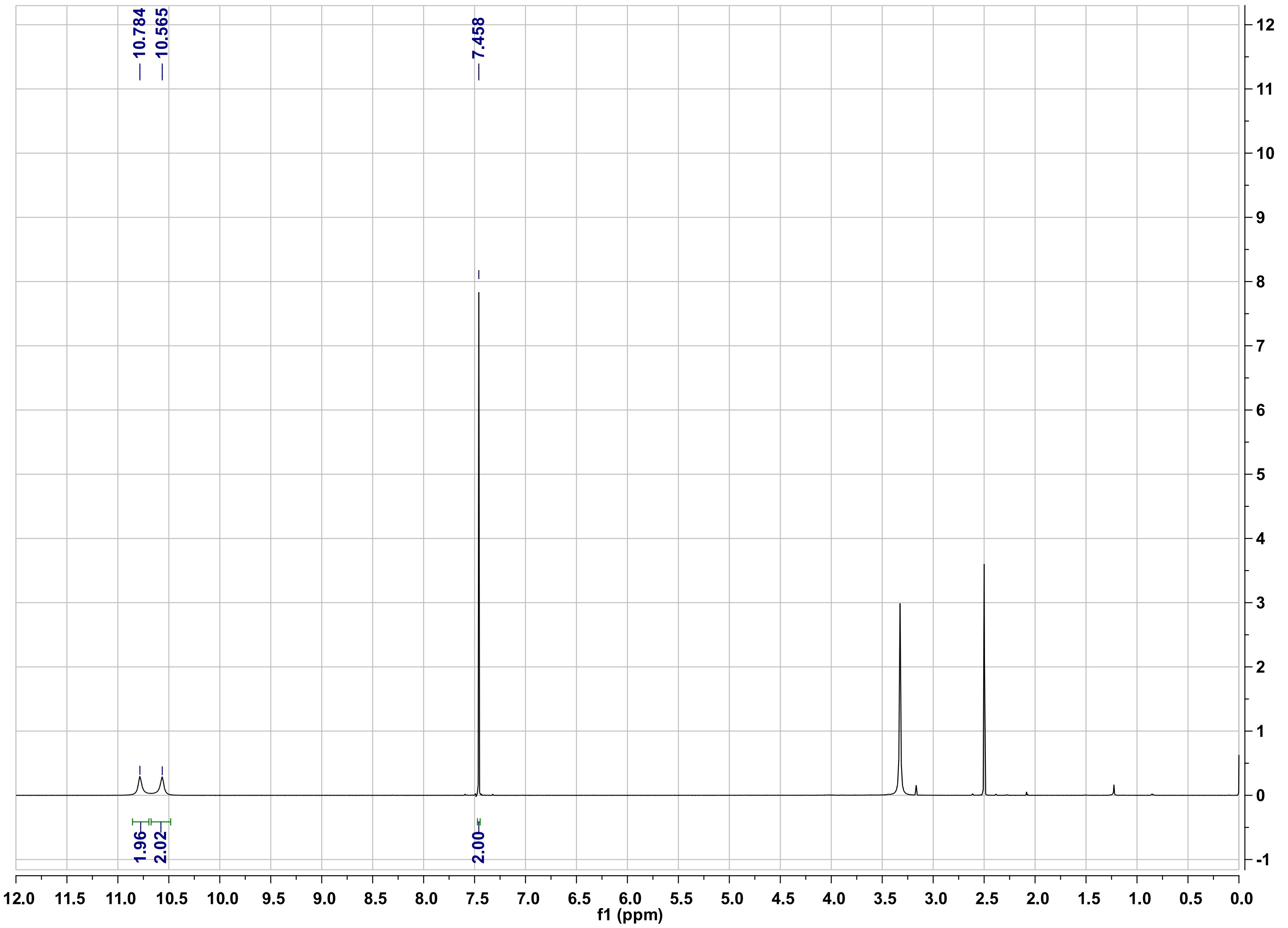

Figure S10. ^{1}H NMR (600 MHz, DMSO-d_6) spectrum of ellagic acid (**compound B**)

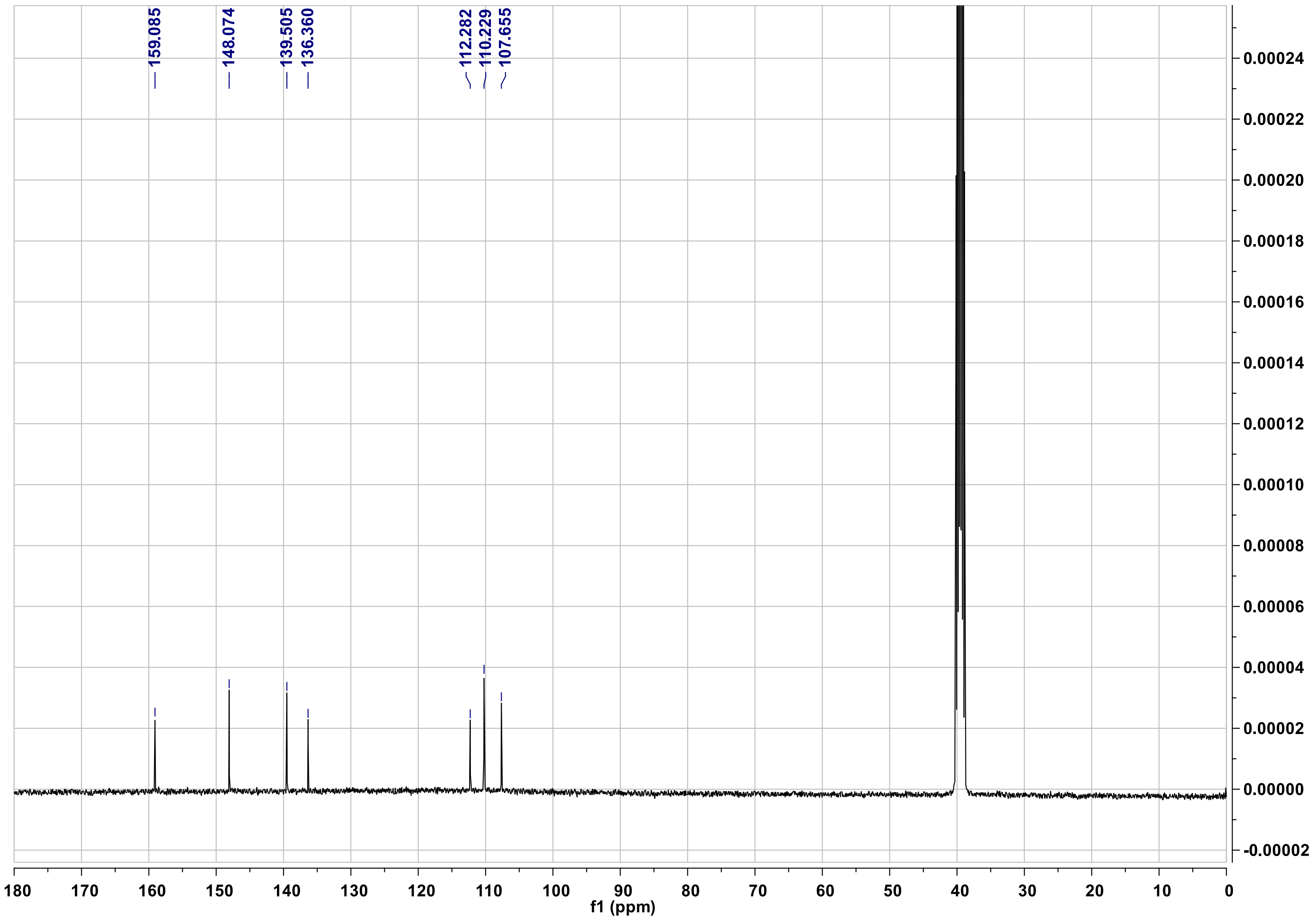

Figure S11. ^{13}C NMR (100 MHz, DMSO-d_6) spectrum of ellagic acid (**compound B**)

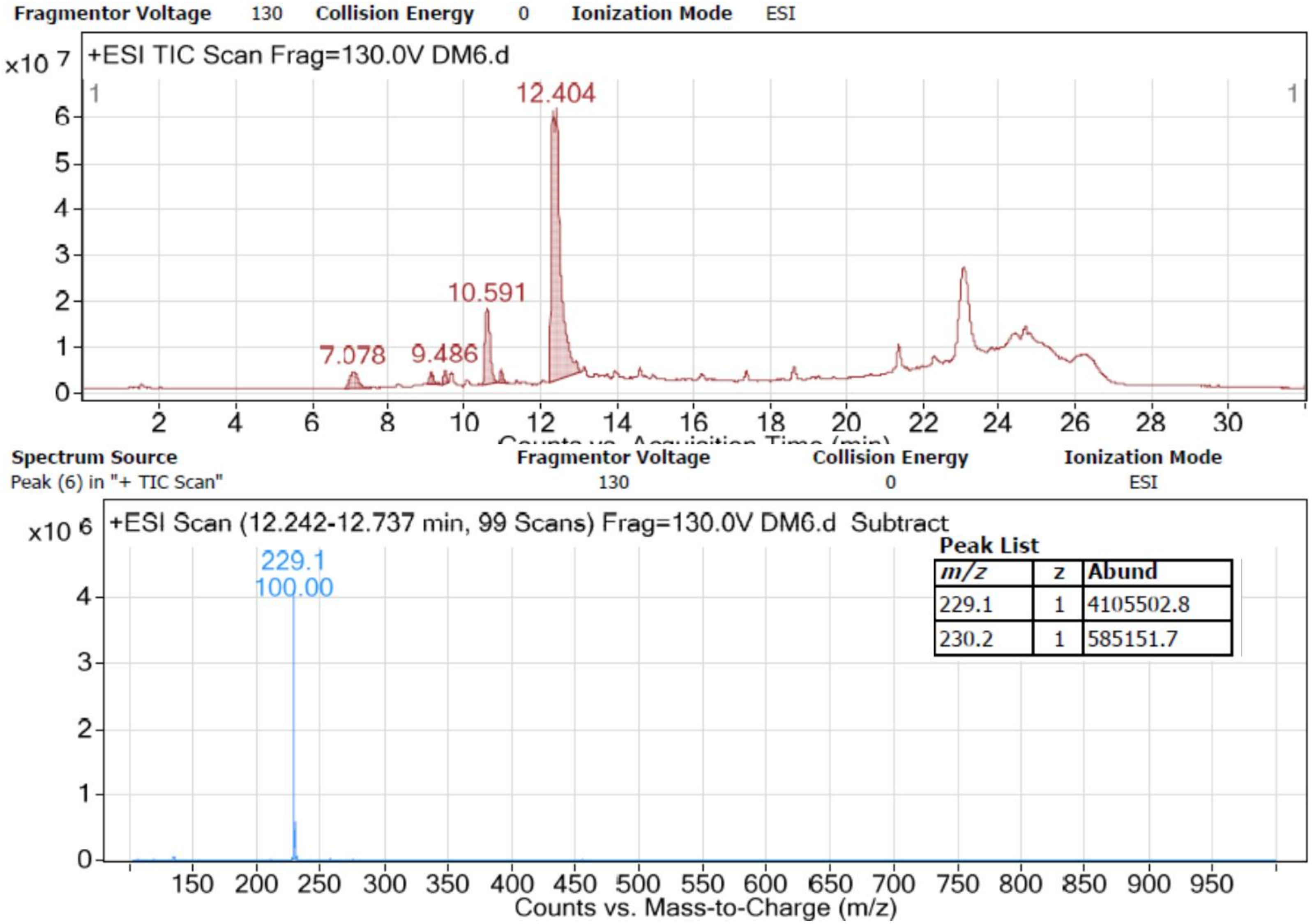

Figure S12. ESIMS spectrum of resveratrol (**compound C**)

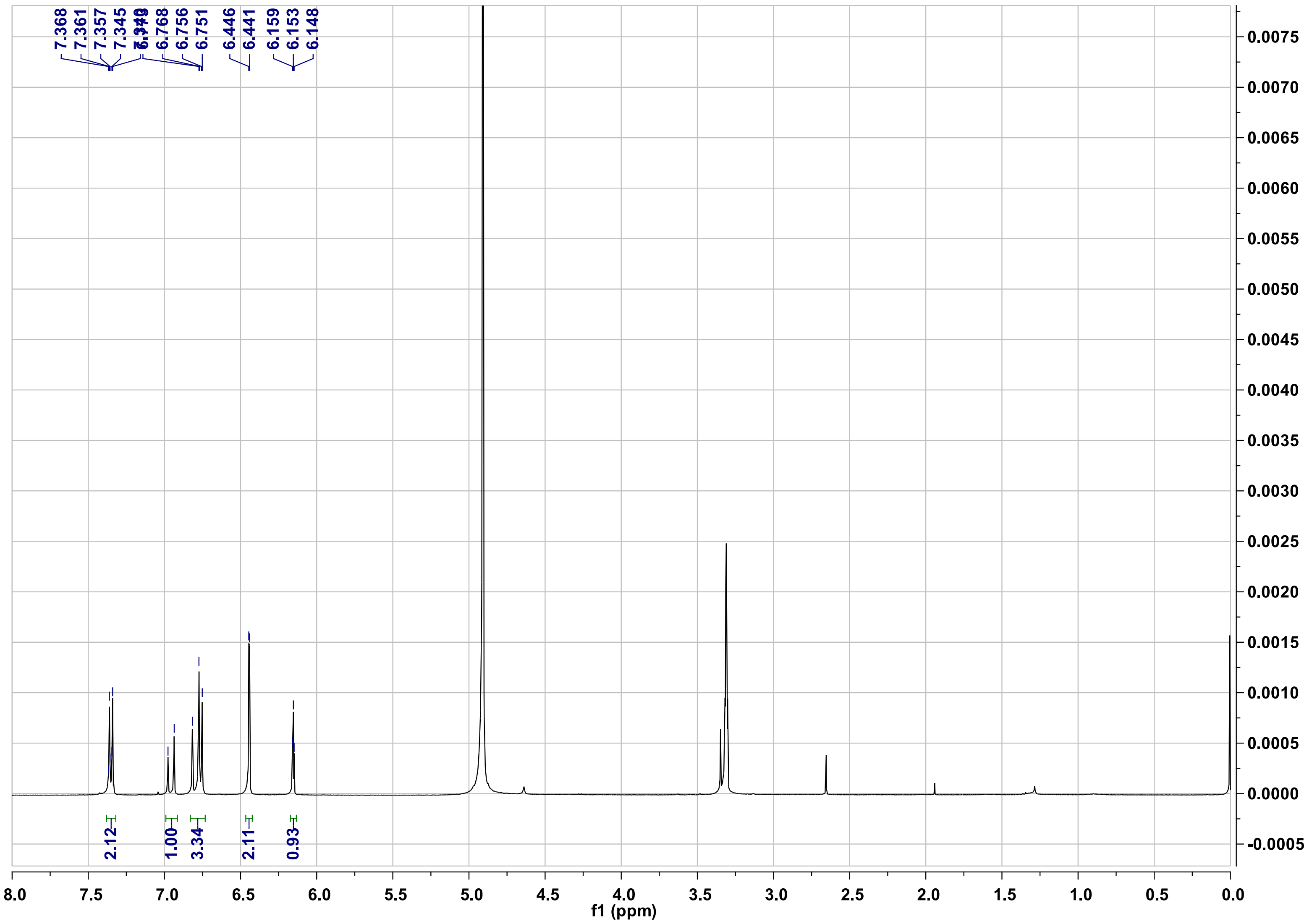

Figure S13. ^{1}H NMR (400 MHz, methanol-*d*$_4$) spectrum of resveratrol (**compound C**)

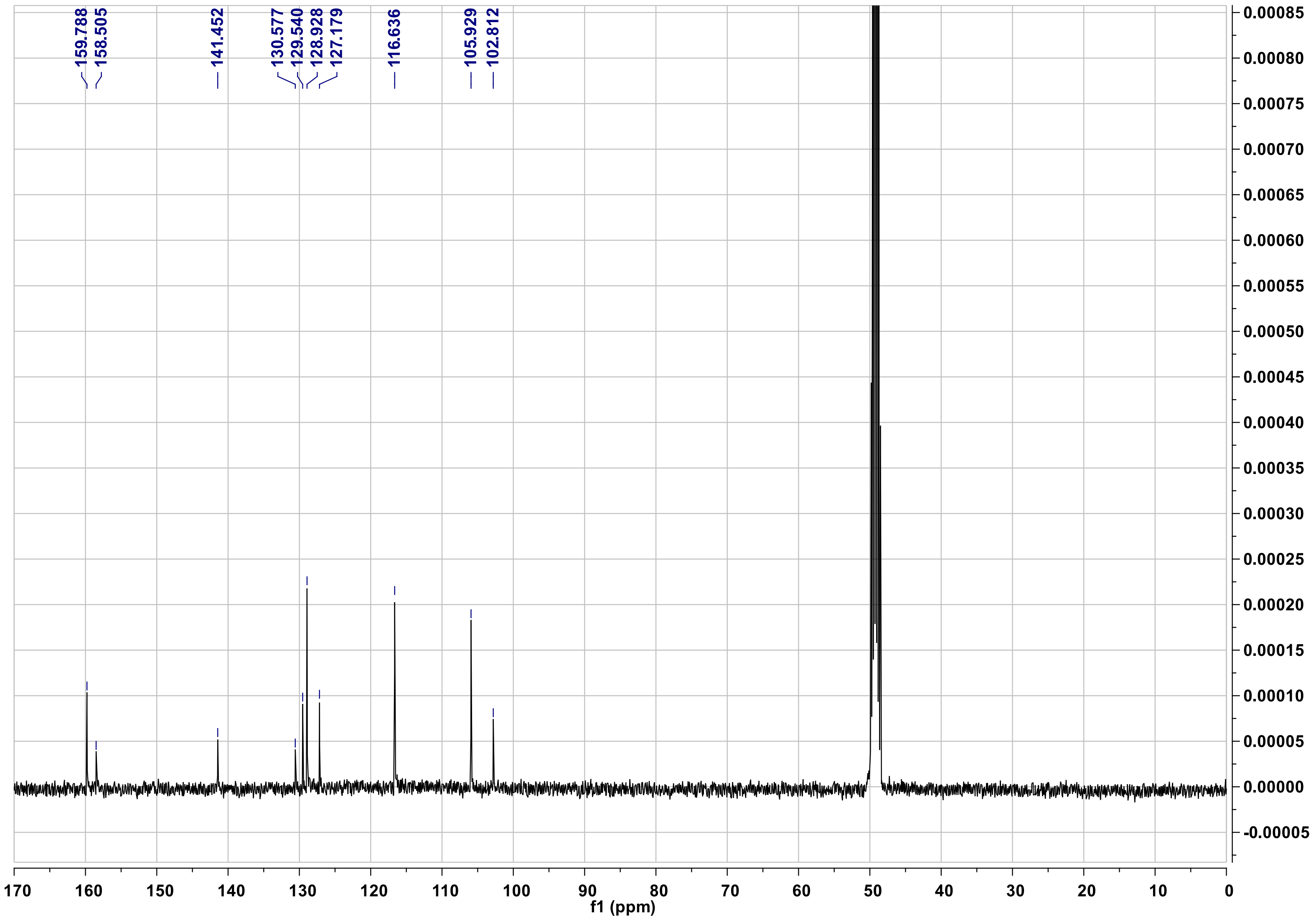

Figure S14. ^{13}C NMR (100 MHz, methanol-d_4) spectrum of resveratrol (**compound C**)

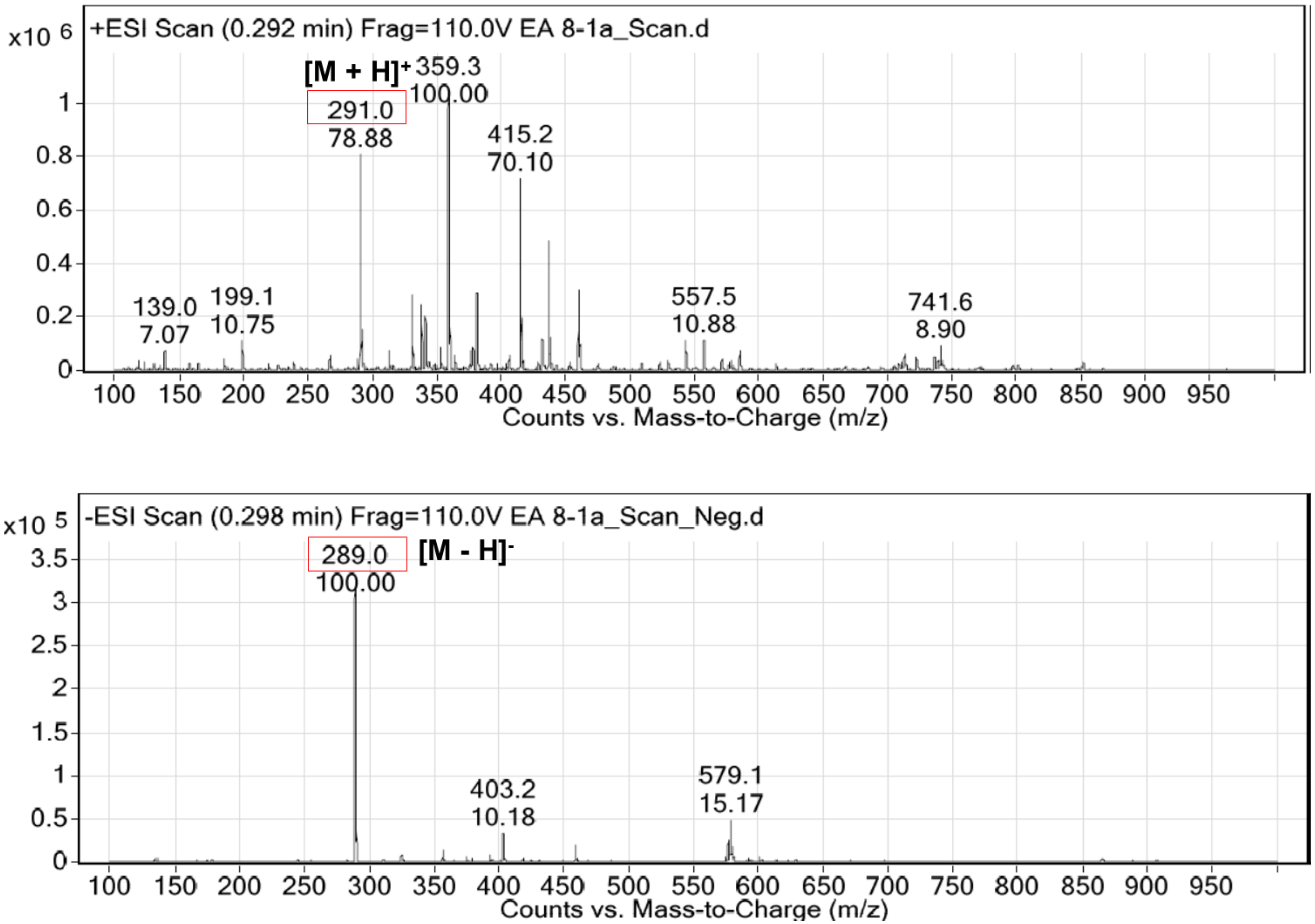

Figure S15. ESIMS spectrum of catechin (**compound D**)

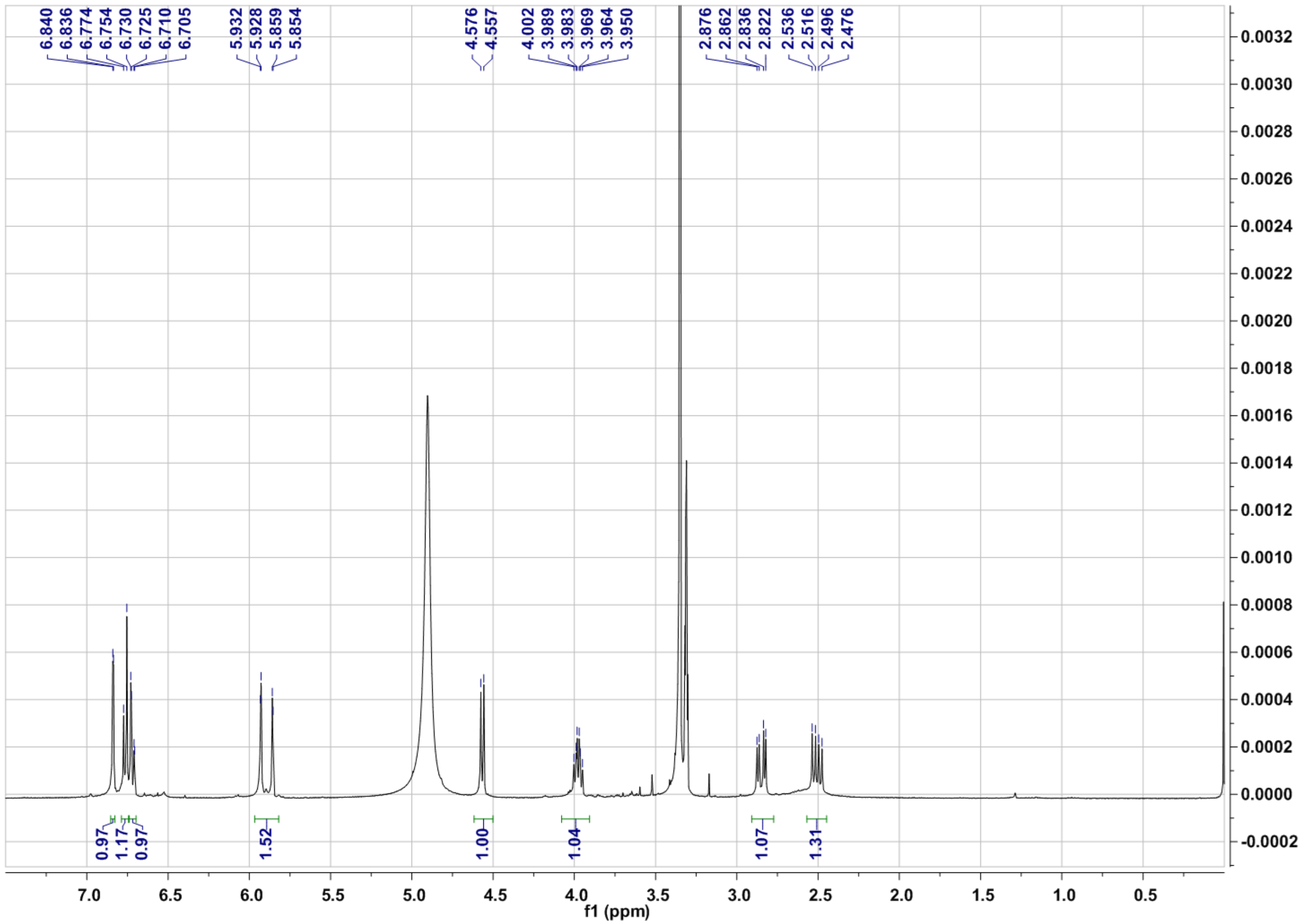

Figure S16. ^{1}H NMR (400 MHz, methanol-d_4) spectrum of catechin (**compound D**)

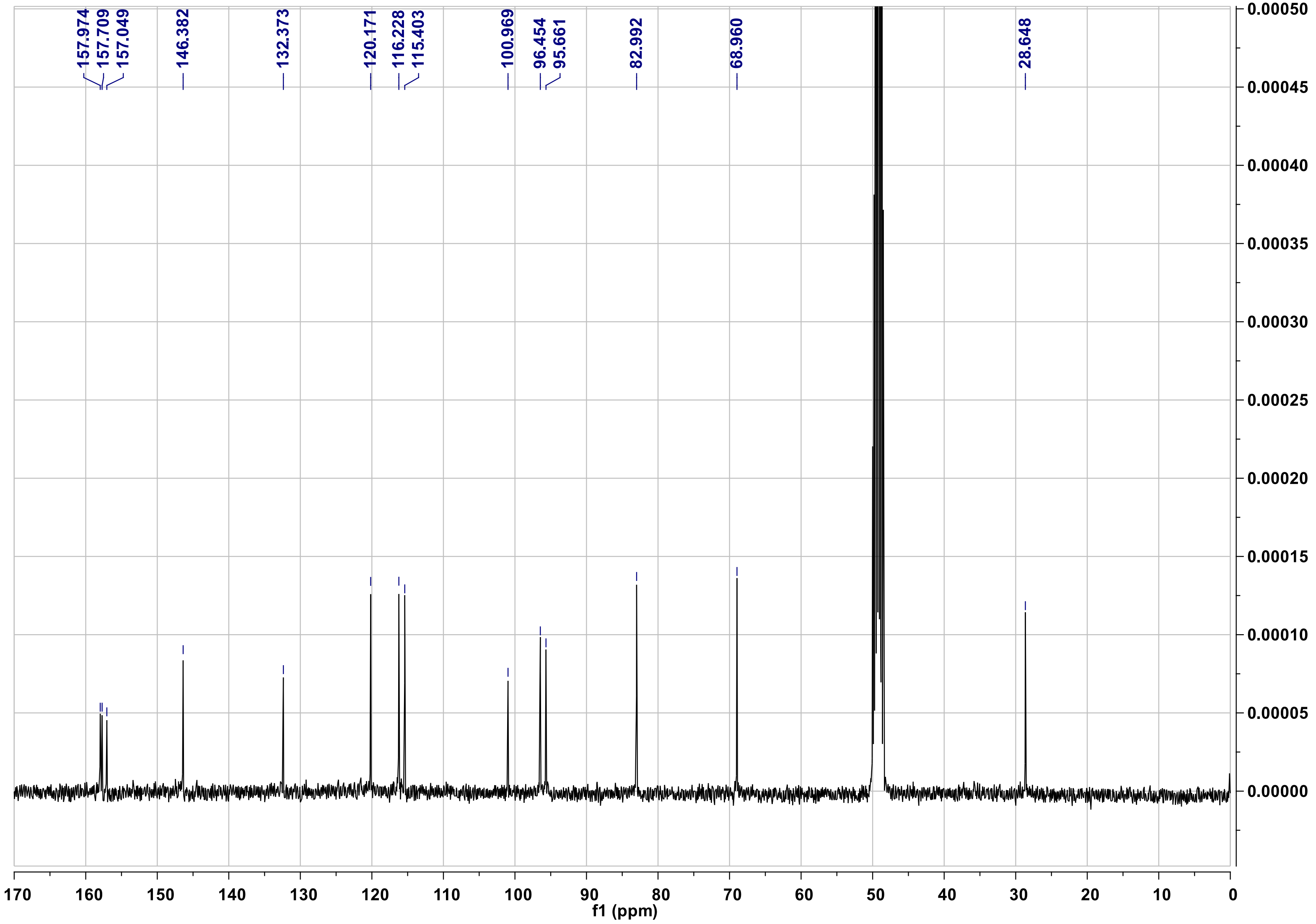

Figure S17. ^{13}C NMR (100 MHz, methanol-d_4) spectrum of catechin (**compound D**)

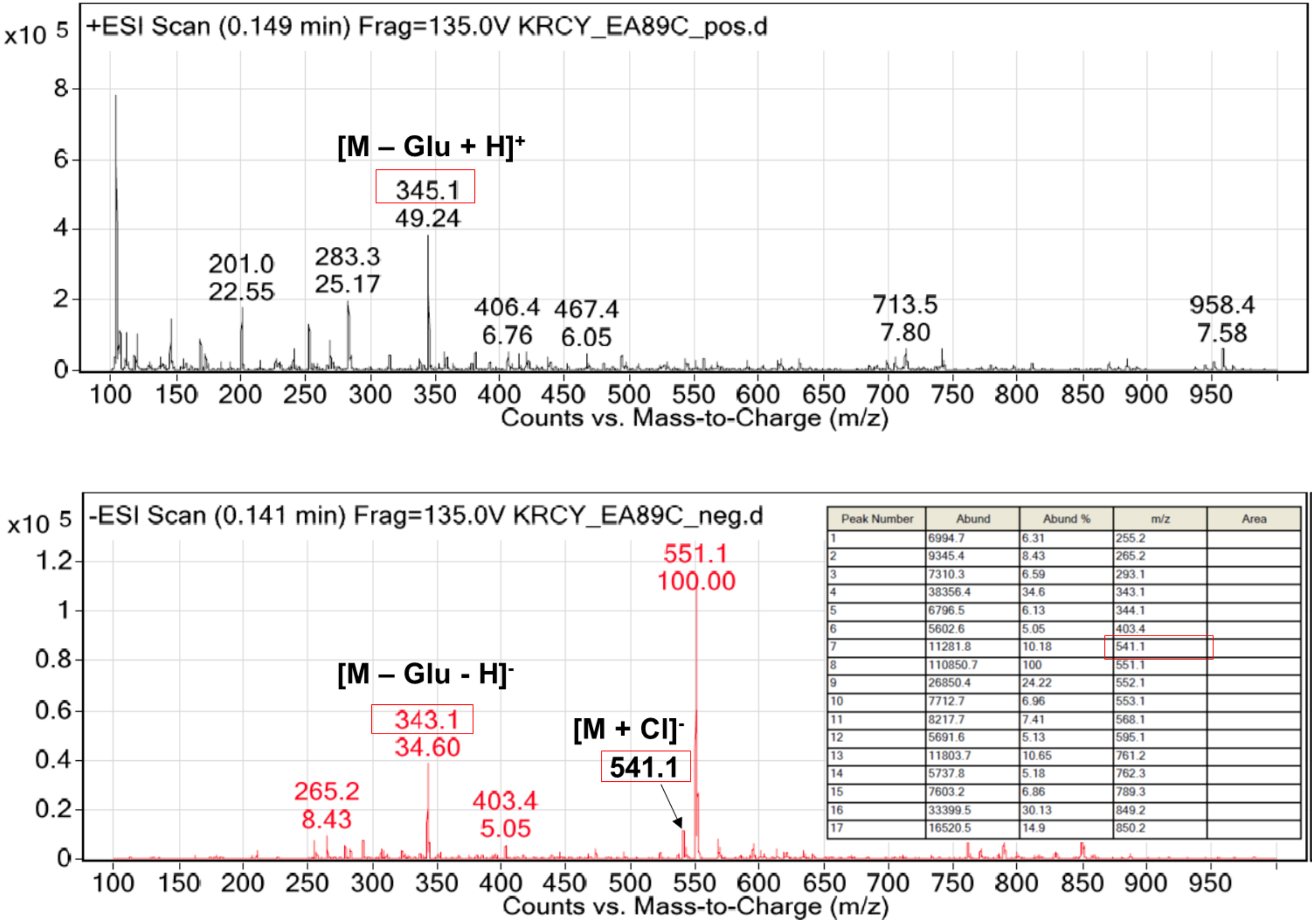

Peak Number	Abund	Abund %	m/z	Area
1	6994.7	6.31	255.2	
2	9345.4	8.43	265.2	
3	7310.3	6.59	293.1	
4	38356.4	34.6	343.1	
5	6796.5	6.13	344.1	
6	5602.6	5.05	403.4	
7	11281.8	10.18	541.1	
8	110850.7	100	551.1	
9	26850.4	24.22	552.1	
10	7712.7	6.96	553.1	
11	8217.7	7.41	568.1	
12	5691.6	5.13	595.1	
13	11803.7	10.65	761.2	
14	5737.8	5.18	762.3	
15	7603.2	6.86	789.3	
16	33399.5	30.13	849.2	
17	16520.5	14.9	850.2	

Figure S18. ESIMS spectrum of 3,3′4′-O-tri-methylellagic acid 4-*O*-*β*-D-glucopyranoside (**compound E**)

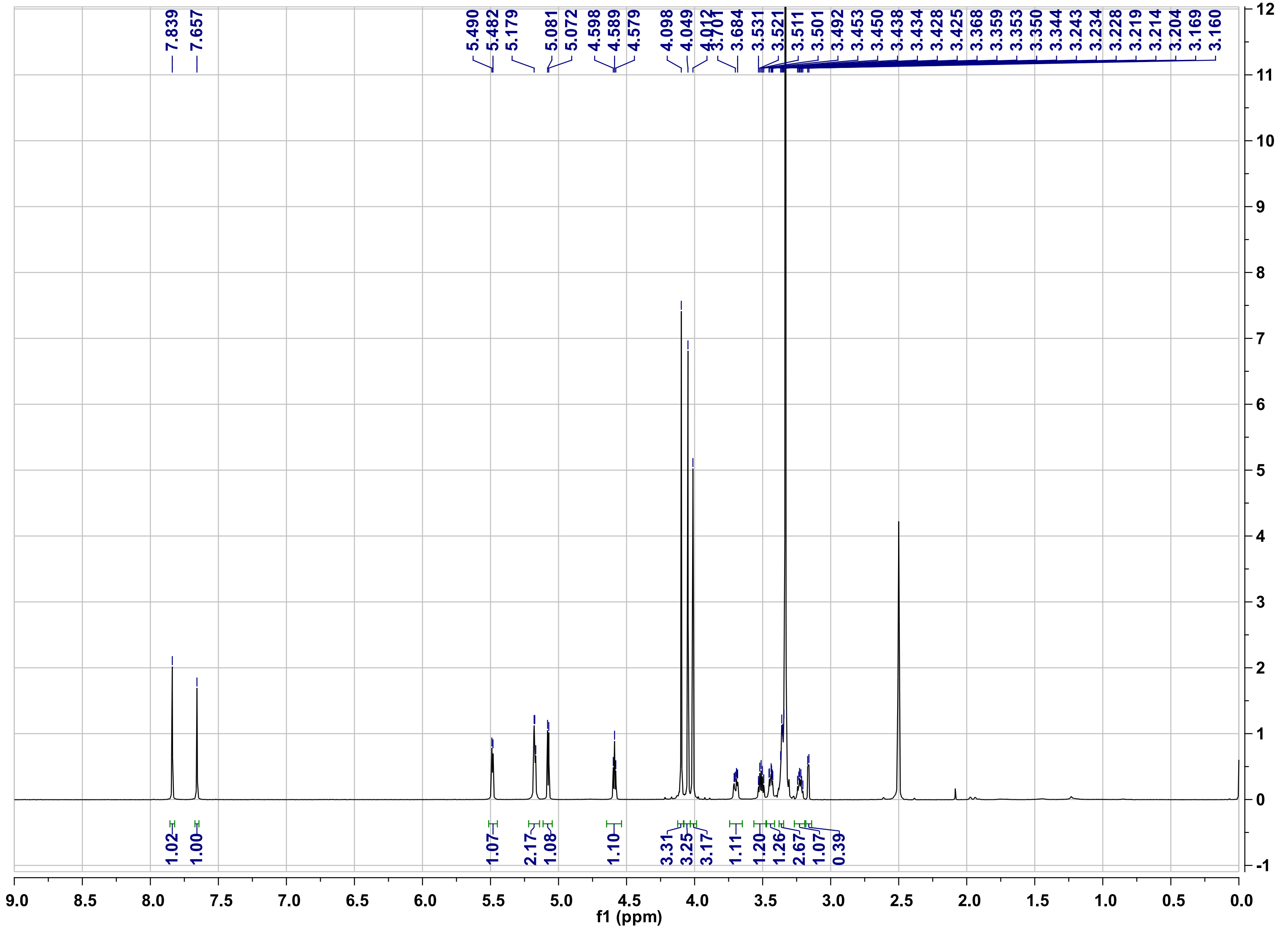

Figure S19. ^{1}H NMR (600 MHz, DMSO-d_6) spectrum of 3,3′4′-O-tri-methylellagic acid 4-*O*-*β*-D-glucopyranoside (**compound E**)

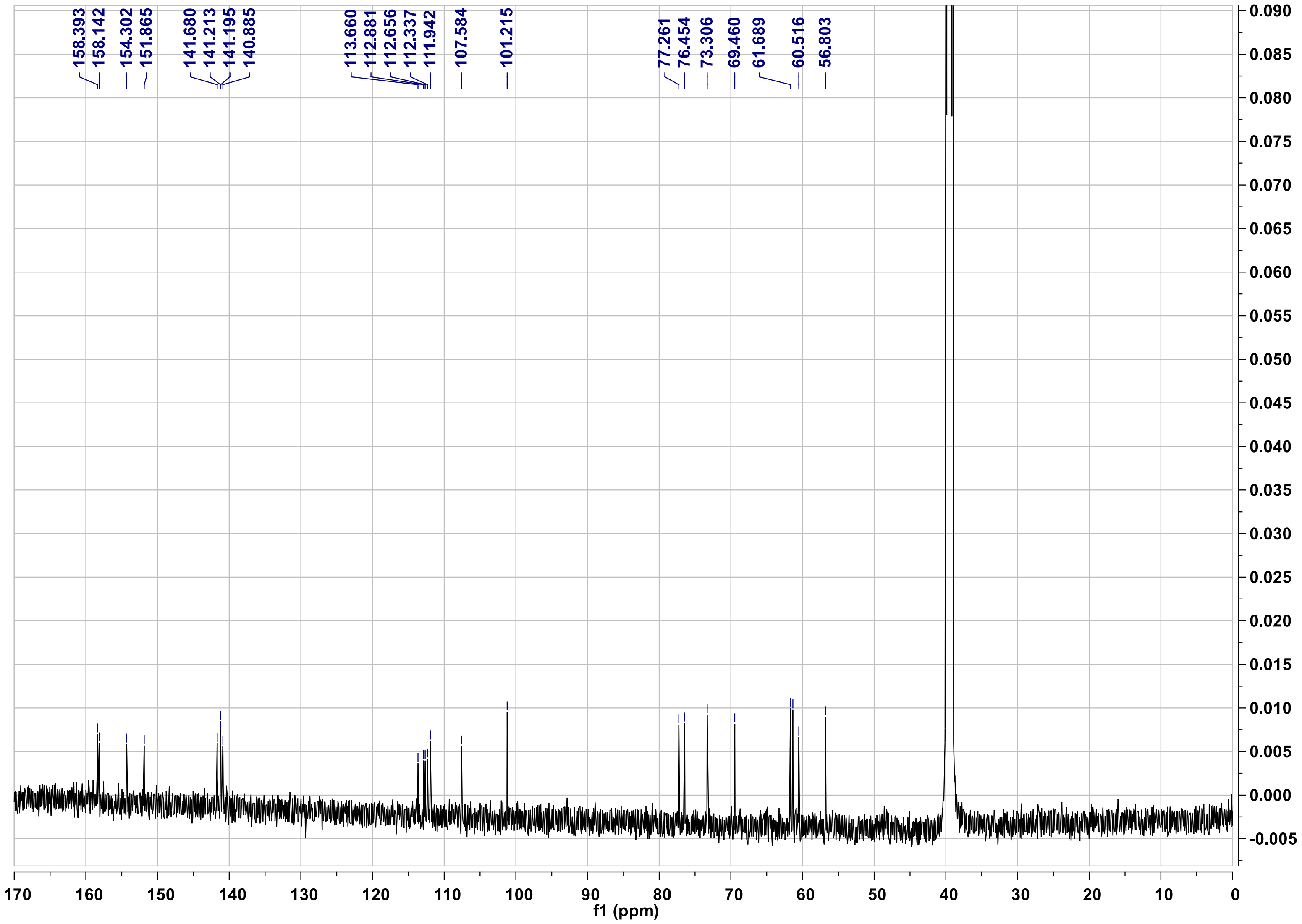

Figure S20. ^{13}C NMR (150 MHz, DMSO-d_6) spectrum of 3,3′4′-O-tri-methylellagic acid 4-*O*-*β*-D-glucopyranoside (**compound E**)
